# Role of Machine Learning Assisted Biosensors in Point-of-Care-Testing For Clinical Decisions

**DOI:** 10.1021/acssensors.4c01582

**Published:** 2024-08-15

**Authors:** Manish Bhaiyya, Debdatta Panigrahi, Prakash Rewatkar, Hossam Haick

**Affiliations:** † Department of Chemical Engineering and the Russell Berrie Nanotechnology Institute, Technion, Israel Institute of Technology, Haifa 3200003, Israel; ‡ School of Electrical and Electronics Engineering, Ramdeobaba University, Nagpur 440013, India; § Department of Mechanical Engineering, Israel Institute of Technology, Haifa 3200003, Israel

**Keywords:** Healthcare, Point-of-Care-Testing, Clinical decisions, Machine learning, Biosensors, Diagnosis, Electrochemical, Lab-on-chip, Electrochemiluminescence, Wearable, Colorimetric

## Abstract

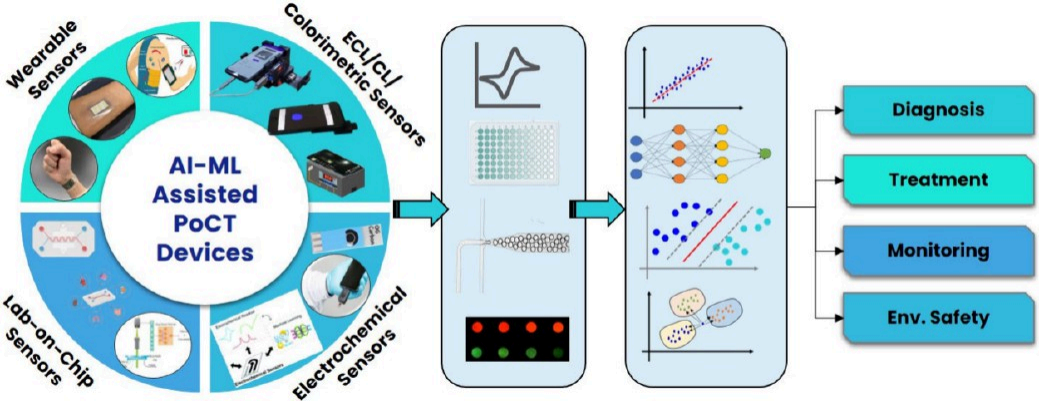

Point-of-Care-Testing (PoCT) has emerged as an essential component of modern healthcare, providing rapid, low-cost, and simple diagnostic options. The integration of Machine Learning (ML) into biosensors has ushered in a new era of innovation in the field of PoCT. This article investigates the numerous uses and transformational possibilities of ML in improving biosensors for PoCT. ML algorithms, which are capable of processing and interpreting complicated biological data, have transformed the accuracy, sensitivity, and speed of diagnostic procedures in a variety of healthcare contexts. This review explores the multifaceted applications of ML models, including classification and regression, displaying how they contribute to improving the diagnostic capabilities of biosensors. The roles of ML-assisted electrochemical sensors, lab-on-a-chip sensors, electrochemiluminescence/chemiluminescence sensors, colorimetric sensors, and wearable sensors in diagnosis are explained in detail. Given the increasingly important role of ML in biosensors for PoCT, this study serves as a valuable reference for researchers, clinicians, and policymakers interested in understanding the emerging landscape of ML in point-of-care diagnostics.

In the recent era, Point-of-Care-Testing (PoCT) has emerged as an essential and transformational part of modern healthcare systems.^1,2^ This paradigm change has been fueled by the urgent need for rapid, cost-effective, and easily accessible diagnostic solutions in a world where healthcare sector is increasingly more decentralized and patient-centric.^3,4^ In addition, PoCT has the promise of allowing healthcare providers and patients to obtain diagnostic information swiftly and comfortably at the point of treatment, whether in a doctor’s office, a distant clinic, or even the comfort of one’s own home.^5−7^

Biosensors are devices that measure and identify compounds using biological component. They aid in the fast and precise detection of particular compounds in fields such as food safety, environmental testing, and healthcare.^8,9^ The use of biosensors in PoCT offers various advantages, such as decreasing the sample volume, conducting screening on-site, and being cost-effective. It also eliminates the need for trained personnel that is required in conventional lab-based testing, which makes it a highly valuable method in different healthcare settings. The integration of biosensors with Machine Learning (ML) has also played a crucial role in the healthcare revolution (Figure 1). This transformation in PoCT delivery can be attributed to several significant factors.^10,11^ First, ML-based biosensors can significantly improve the sensitivity, accuracy, and efficiency of PoCT sensors. These algorithms are capable of processing enormous quantities of complex biological data, which enables biosensors to precisely detect and interpret even the smallest changes in biomarkers.^12,13^ This enhanced sensitivity is essential for the early detection of diseases, enabling medical professionals to identify issues at the best moment for intervention. Moreover, the precision offered by ML algorithms diminishes the rates of false positives and negatives, decreasing the possibility of incorrect diagnosis and, consequently, needless medical interventions or missed conditions. Such precision is particularly crucial in cases where the outcomes of diagnostic tests constitute a major factor in healthcare decisions. Second, the quick data processing capabilities of the ML based biosensors represent another important advantage. When it comes to PoCT, time is frequently of the essence, whether it be during routine patient visits, infectious disease treatment, or medical emergencies.^14,15^ Biosensors driven by ML provide results in almost real-time, facilitating prompt clinical decision-making and more effective patient care. Third, ML applications in biosensors extend beyond sensitivity and accuracy. The ML technology is versatile and adaptable, offering remedies for a variety of diagnostic problems. The diagnostic powers of biosensors are certainly enhanced by ML models, which classify intricate data sets, carry out regression analysis, and spot patterns and trends in big data sets. Finally, the integration of ML with biosensors for PoCT enhances the precision and effectiveness of diagnosis while enabling real-time monitoring, individualized treatment, and prompt responses to medical emergencies. It is a cornerstone of the future of healthcare, where technology and medicine meet to improve patient outcomes and overall healthcare quality.^16,17^

**Figure 1:**
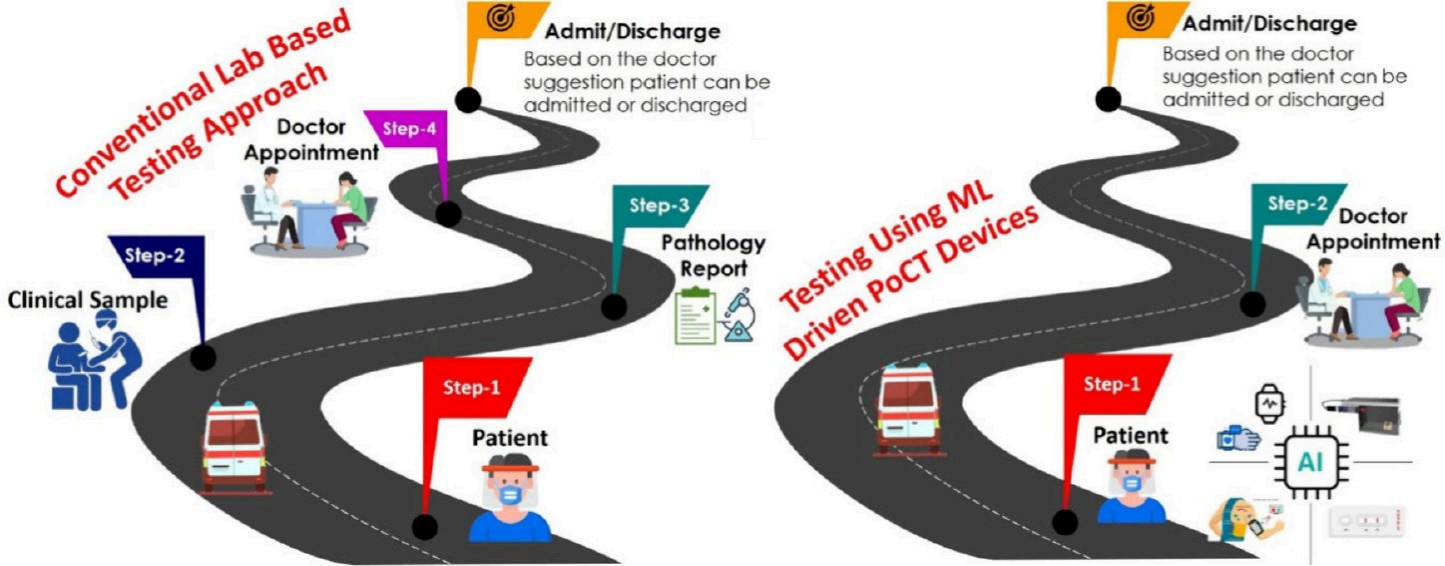
Steps involved in conventional laboratory-based testing and PoCT.

This review aims to highlight the detailed working principles of several prominent regression- and classification-based ML algorithms, examining their applicability in predicting different analyte disease biomarker specifications. The objective is to focus on the comparative analysis of ML models in the context of PoCT, offering a detailed examination of their practical applications and performance metrics. Moreover, it also intends to provide a comprehensive overview of emerging trends and future directions in ML-assisted biosensor technology. The article is organized into the following sections: Supervised ML and Its Broad Categories briefly explains the working principles of different ML models; Application of ML for PoCT explores the application of ML in the field of PoCT, followed by the conclusion. Figure 2 illustrates the ML process flow, outlining the pathway from sensing and data collection to data analysis with ML algorithms and applications.

**Figure 2:**
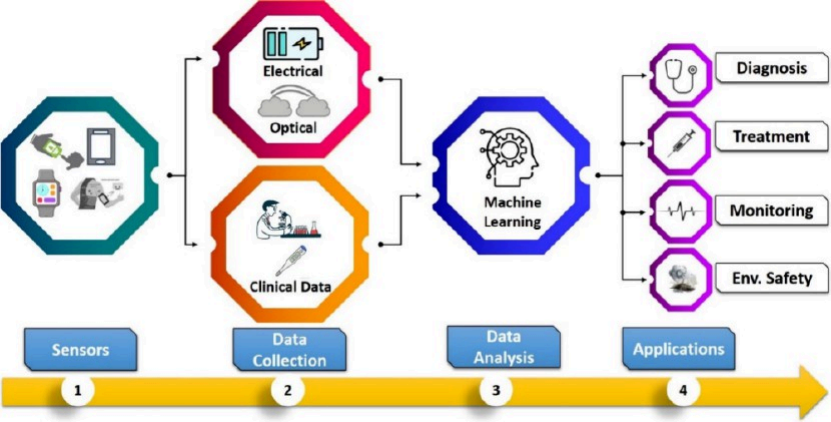
Depicts a visual representation of the ML process flow, which includes the pathway from sensing and data collection to analysis with ML algorithms and applications.

## Broad Classification of ML

**Figure 3:**
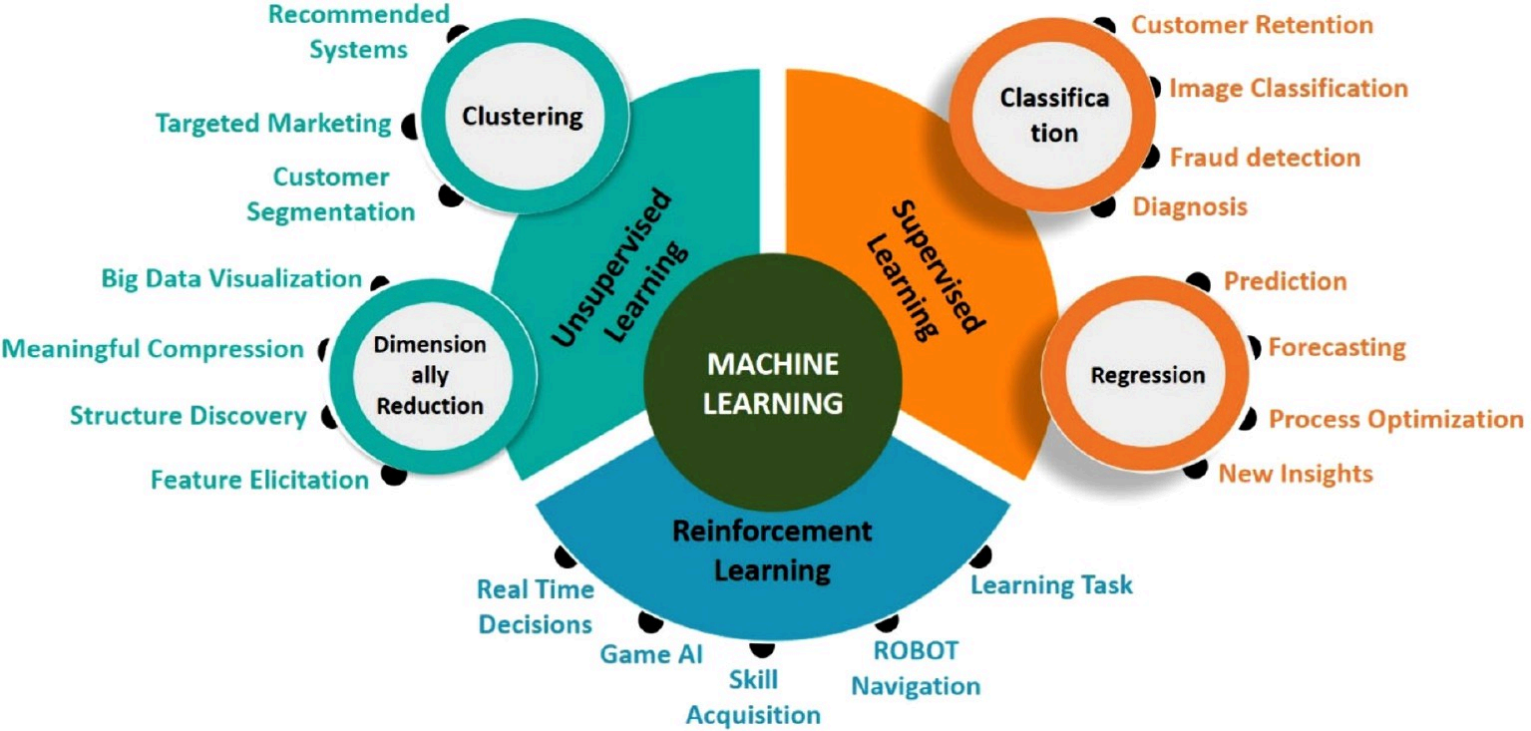
Hierarchical Insight: Tree diagram illustrating ML paradigms and their diverse types.

ML is typically classified into three types: supervised learning, unsupervised learning, and reinforcement learning. Each type serves a particular purpose and is suitable for different types of applications.^18^ This review article covers an in-depth understanding of supervised ML models and their diverse applications in the field of PoCT. Figure 3 shows the comprehensive tree diagram illustrating the ML paradigms and their diverse types.

**Figure 4:**
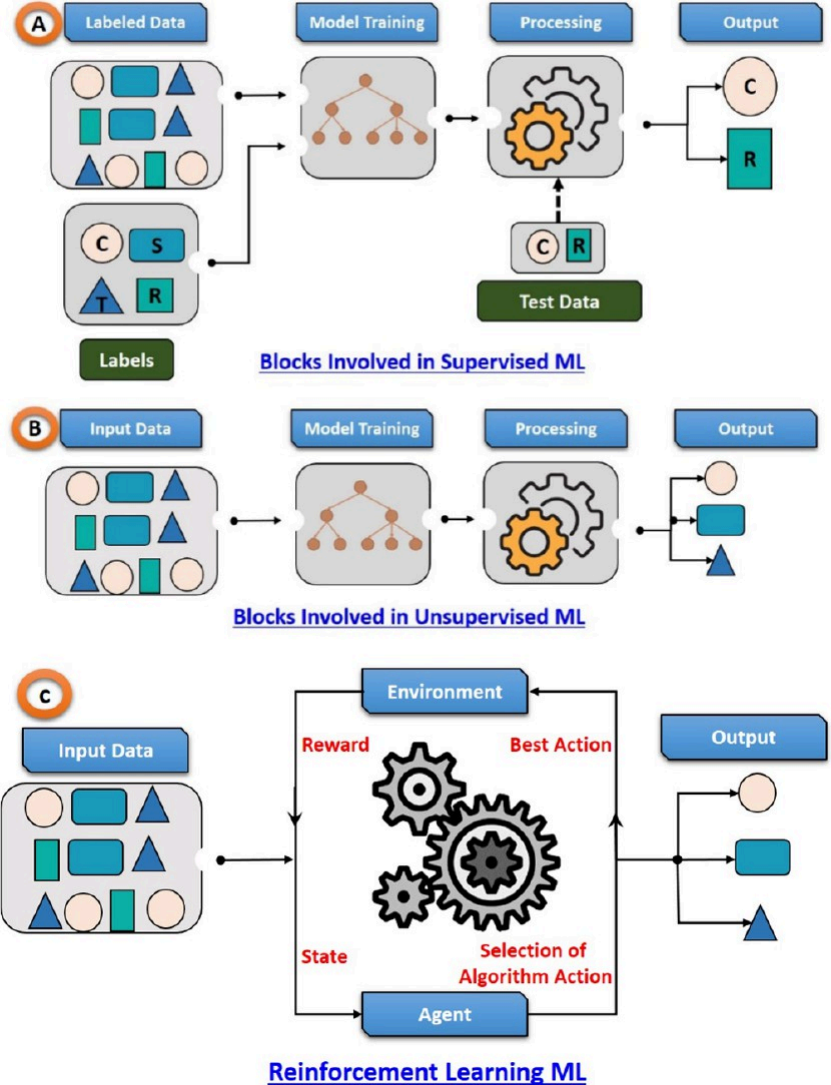
Working principle and various blocks involved in Supervised/Unsupervised/Reinforcement ML. (A) Various block involved in supervised ML. (B) Various block involved in unsupervised ML. (C) Various blocks involved in reinforcement ML.

Supervised ML is a type of ML in which the algorithm is trained on a labeled data set, which means that the input data is paired with corresponding output labels, shown in Figure 4A. The purpose of supervised learning is to train a function that maps input variables to desired output variables.^19,20^ Unsupervised learning (Figure 4B) is the process of training a model using a data set that does not contain labeled responses. The model attempts to identify patterns or structures in the input data such as clusters or associations. In case of, reinforcement learning, it involves training a model through interactions with an environment, Figure 4C. The model learns a policy that maximizes cumulative rewards by taking actions and receiving feedback in the form of rewards or penalties.^21^

To this end, ML algorithms have the potential to improve PoCT device performance in a number of ways, including lowering processing times, increasing diagnostic accuracy, and enhancing sensitivity and specificity.^22^ Among three, supervised learning models provides good sensitivity, specificity, and accuracy by carefully training on labeled data sets, where each input (features) is paired with the correct output (label). This makes it possible for the model to precisely understand the relationship between inputs and outputs. Sensitivity (true positive rate) is improved by training the model with a diverse set of positive cases, allowing it to identify patterns linked to these cases effectively. Specificity (true negative rate) is increased by ensuring that the model is trained with sufficient negative cases, helping it distinguish between positive and negative outcomes accurately. Finally, learning both positive and negative situations improves accuracy, the model’s overall correctness, and improves overall prediction ability of the supervised ML models.^23,24^

In addition, supervised ML models provides better performance as compared to others because of quality and diversity of training data, feature selection and engineering, the choice of algorithm and its optimization, and regular evaluation and validation of the model through techniques like cross-validation, threshold adjustment, and hyper parameter tuning.^25^Table 1 presents a comparative comparison of supervised, unsupervised, and reinforcement learning techniques in the context of PoCT devices.

**Table 1:** Comparative Analysis between Supervised, Unsupervised, And Reinforcement Learning Techniques^26−28^

**Parameter**	**Supervised ML Model**	**Unsupervised ML Model**	**Reinforcement ML Model**
**Accuracy**	Diverse and comprehensive data set is available leading high accuracy	Provides moderate accuracy as it completely depends on the complexity of data pattern	If perfectly designed rewards system is available which turns high accuracy
**Specificity**	If the data set is balanced then it will provide high specificity	If labels are not properly differentiate resulting lower accuracy	If the false positive are penalized then it will provide high level of accuracy
**Sensitivity**	Provides high sensitivity because of availability of labeled data set	Provides lower sensitivity as compared to other twos because it is unable to identify positives	If the well-defined reward structure is available then it can provide high accuracy
**Processing Time**	It completely depends on data set size and complexity of algorithm	It can vary based on the applications	Need lot of time during training phase but consumes less time in real time
**Complexity**	Completely depend on model used	High, as it need to find out the hidden pattern in data set	High, due to iterative learning process

## Supervised ML and Its Broad Categories

### Types of Supervised ML

Based on the nature of the output variable, supervised ML can be classified into two categories: regression and classification. In regression, the algorithm is trained on a data set with a continuous output variable. The purpose is to learn mapping from inputs to predict a numeric value. On the other hand, in classification, the algorithm is being trained on a data set where the output variable is a category or label.^29^ The goal is to learn and find mapping from inputs and to provide predictions in terms of discrete classes or categories.

### Performance Matrix for Regression and Classification Supervised ML

In order to ensure that PoCT devices provide reliable, precise, and timely healthcare information, performance measures for regression and classification in supervised ML are essential for device development and validation. The performance measures used in regression and classification operations are determined by the nature of the problem and the specific objectives of the investigation. Here are some standard performance metrics for regression and classification.^30,31^ In the case of regression, the performance of the ML models can be evaluated using the following metrices: Mean absolute error (MAE), mean squared error (MSE), root mean squared error (RMSE) and R-2 score.

### Regression Performance Metrics


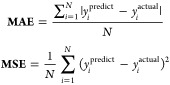



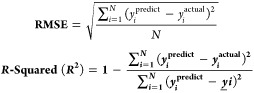


### Classification Performance Metrics


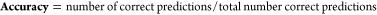



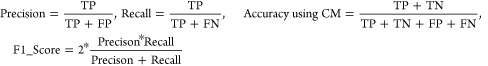

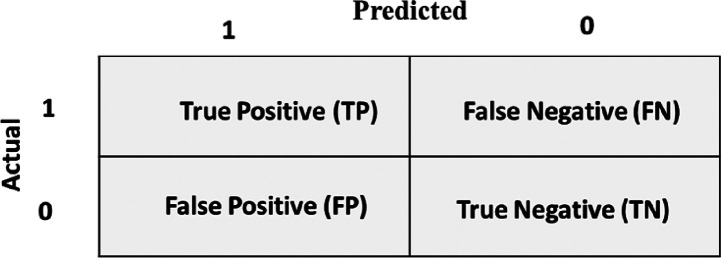




In an ideal case, a well-performing model exhibits minimal error values and a maximized R-squared score. The average absolute difference between expected and actual values is calculated using the MAE. MAE and the target column both have the same output column. Its computation requires the modulus function, which is nondifferentiable at *t* = 0, which poses a considerable challenge. This issue prompted the introduction of the MSE. The MSE determines the average squared difference between actual and predicted values. The unit of MSE is square of the output column, potentially causing confusion due to larger numerical values. This concern is addressed by the RMSE, which shares the same unit as the output column, offering a more understandable measure. *R*^2^ is also called as goodness of fit and its value ranges from zero to one. Ideally, the *R*^2^ value should be as high as possible, approaching toward 1 which indicates the model is performing well. In case of regression, it is difficult to compute performance based on single matrix. Hence, to establish concreate results it is good to compute all those four important performance matrices.

In the classification scenario, the performance of the ML models can be evaluated using the following metrices: Accuracy, precision, recall, and F1 score. Accuracy can help to determine the overall correctness of the model in predicting classes. It is defined as the ratio of the number of correct predictions to the total number of predictions. Precision measures the accuracy of positive predictions. It is the ratio of correctly predicted positive observations (TP) to the total predicted positives (TP + FP). Recall measures how well the model can identify and include all pertinent instances. It is the ratio of correctly predicted positive observations (TP) to all of the actual positives (FN). Finally, the F1 score represents the harmonic mean of precision and recall. If there is unequal distribution of data, that time F1 score plays an important role. These are the few most important matrices in the case of classification to measure the performance of the model.

### Exploring the Working Principle of Most Reported ML Algorithms

Within the broad field of ML, a wide range of models has emerged, each with distinct operational concepts and techniques. To successfully navigate the challenging field of biosensors and choose the optimal algorithm for a specific task, one must have a thorough understanding of the nuances of these models. Every approach, from advanced neural networks to conventional models such as linear regression, is intended to address particular problems and trends in the data. This section explores the working principles of various ML models aims to provide their functionality, laying down the foundational understanding for enthusiasts, early career researchers, and decision-makers venturing into the dynamic realm of ML and predictive analytics.

### Linear Regression

**Figure 5:**
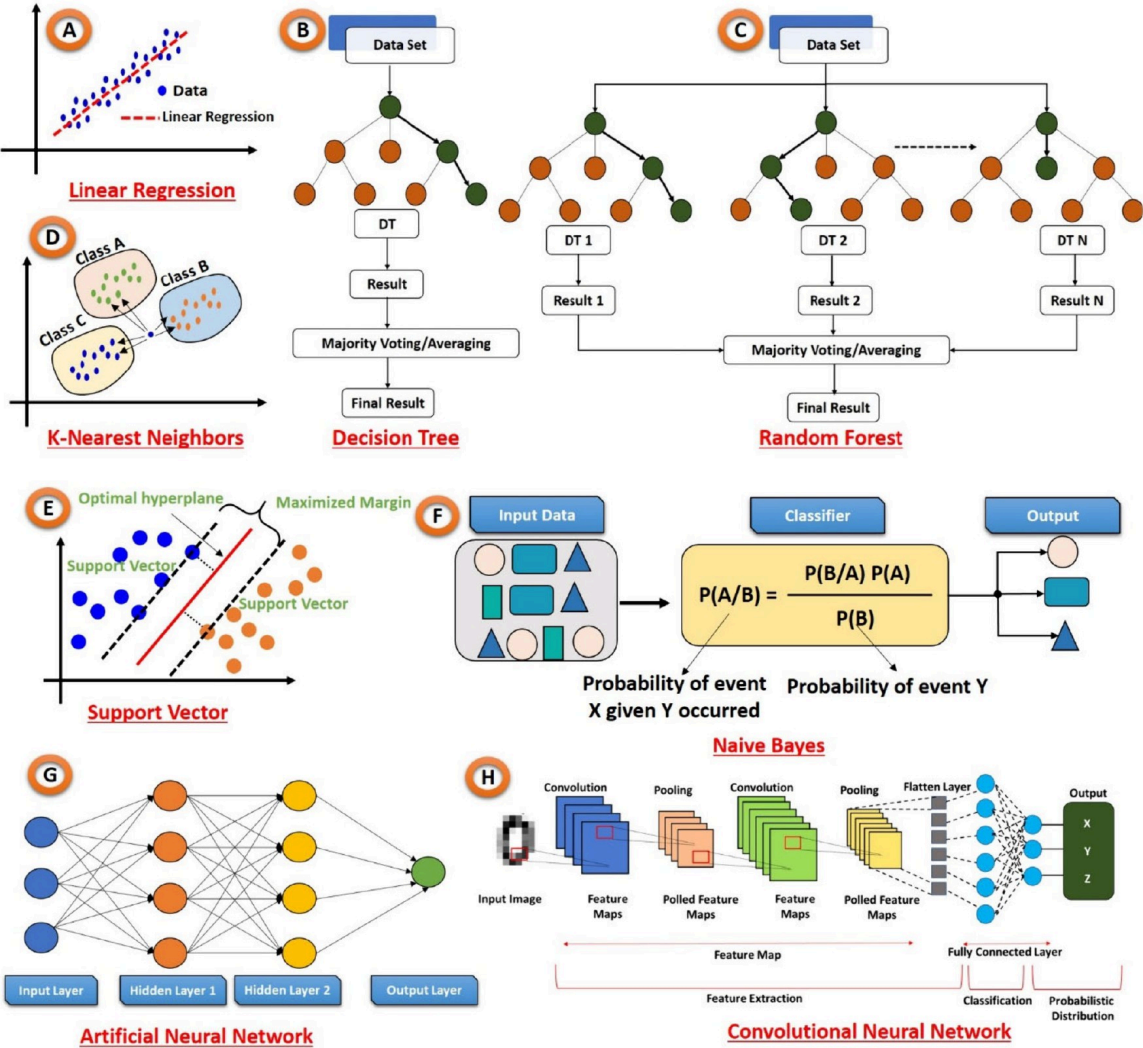
Working concept for different supervised ML models: (A) Linear regression, (B) Decision tree, (C) Random forest, (D) k-Nearest neighbor, (E) Support vector, (F) Naive bayes, (G) Artificial neural network, and (H) Convolutional neural network.

Consider a graph with several data points (shown in Figure 5A). The straight line that most closely fits these points can be found by using linear regression. You can use this line to predict outcomes or comprehend how different factors relate to one another. The line that best-fits these data points most accurately is found using linear regression.^32,33^ The term “best fit” refers to how closely the line matches each dot. The straight line that matches each data point can be written as *y* = *mx* + *c*. Where *y* = dependent variable, *x* = independent variable, *m* = slope of the straight line, and *c* = constant.

### Decision Tree

Decision tree is a combination of multiple if-else conditions (shown in Figure 5B), and it can profoundly be used for both regression as well as classification. The following are the steps involved to understand the working principle of decision tree algorithm.^34^ 1) Root node: it represents the entire data sets. 2) Feature selection: The algorithm, based on its values, selects a feature from the data set and splits the data into subsets. The goal is to choose best features from the data sets that result in the most homogeneous subsets in terms of the target variable. 3) Decision node and branches: The chosen feature becomes a decision node. Based on the feature values the tree branches come out into different path. Each branch represents a decision or a condition depending on the selected feature. 4) Nodes: The meeting point of branches is called the node. Based on the requirements fulfilled so far, they serve as a decision point. 5) Stopping criteria: The data are split by the algorithm repeatedly until a predetermined stopping condition is satisfied. A minimal number of samples in a node, maximum tree depth, or a particular degree of subset homogeneity could all be considered stopping criteria. 6) Leave nodes: The final nodes are called leave nodes. They describe the final decision or outcome. Once the stopping criteria are met, in the case of the classification problem, the majority class in a leaf node is assigned as the predicted class. In terms of regression analysis, the average or weighted average of the target variable in the reached leaf node was considered as a predicted value.

### Random Forest

The random forest uses multiple decision trees (shown in Figure 5C) and uses an ensemble learning method for prediction. Ensemble methods in ML uses multiple base models to create an optimal predictive model. Ensemble technique is commonly used to mitigate overfitting issues, where a model performs well on the training data set but underperforms on the testing data set.^35,36^ Ensemble methods use both bagging (a parallel approach) and boosting (a sequential approach) techniques to improve the model performance. The random forest ML model uses multiple decision trees during training and uses voting or averaging concept for prediction in terms of classification and regression, respectively. In the case of classification, for a new data point, each decision tree in the forest predicts a class. The most frequently occurring class was considered as the final predicted output. On the other hand, in the case of regression, for a new input, each decision tree predicts a numerical value. The final prediction is the average of all of the individual decision tree predictions.

### Adaptive Boosting (Ada Boost)

AdaBoost, also known as adaptive boosting, is an ensemble learning method that builds an effective and accurate model by combining the predictions of weak learners, usually decision trees. Mainly, the AdaBoost model used a sequential ensemble model to convert weak learners into strong learners. The sequential ensemble method uses a weighting technique. For each training data point, a specific weight is assigned. Next, across the learning process, these weights are utilized to train each hypothesis. The goal of assigning weights is to give more importance to the incorrectly classified data by assigning more weights, while giving less significance to correctly classified data by assigning it a lower weightage.^37,38^

### Gradient Boost

An ensemble learning approach called gradient boosting can be used for both regression and classification problems. Gradient Boosting is based on the successive construction of a number of weak learners, usually decision trees. Each decision tree corrects the errors of its predecessors, finally leading to the building of a strong predictive model. The additive AdaBoost model identifies residuals from previous models by assigning high weights to data points with higher error rates. Gradient boosting, on the other hand, uses the gradient to identify errors from earlier models. All decision trees in gradient boosting have the same weight, and in order to improve the overall performance of the model, a learning rate limits their predictive influence.

### k-Nearest Neighbors (KNN)

The k-Nearest Neighbors (KNN) algorithm is a simple and distance based powerful ML algorithm used for both classification and regression tasks, as shown in Figure 5D. In the case of regression, the kNN model makes predictions through the following steps. It starts by selecting the K neighbors (k) and uses either the Manhattan distance or the Euclidean distance to calculate the distance between the test and training data points. The closest data points are identified by sorting the computed distances in ascending order. In order to predict the value that corresponds to the test data point, the model last computes the mean. On the other hand, in case of classification, it assigns the class label to the new data point based on the majority class among its k-nearest neighbors. The most frequent class in the neighborhood “votes” for the classification.^39,40^

### Support Vector Machine (SVM)

Support Vector Machines (SVM) are versatile ML models used for both classification and regression tasks. Following are the steps involved to understand the working principle of decision SVM model (shown in Figure 5E). 1) Input data: Receives training data points each labeled with its class. 2) Feature Space Transformation: Transform the input data points into a space with more dimensions. A kernel function is utilized to accomplish this modification. 3) Identify Hyperplane: Locate the hyperplane in the transformed space that most effectively divides the data into different classes. The goal of selecting this particular hyperplane is to maximize the margin the distance between the hyperplane and the closest data points, or support vectors. 4) Handling Nonlinearity: The kernel functions of SVM allow it to implicitly map the data into a higher-dimensional space, allowing it to handle nonlinear correlations between features. 5) Prediction: in the classification scenario, for a new data point, it determines on which side of the hyperplane it falls. The side of the hyperplane determines the class that is assigned. And in the case of regression, it predicts the numerical value based on its position relative to the hyperplane.^41^

### Naive Bayes

Naive Bayes is a simple and powerful ML algorithm that uses the concept of the Bayes theorem. The classification principle of Naive Bayes ML models is shown in Figure 5E. Its fundamental idea is based on probabilistic categorization, especially for predicting the probability of a given sample belonging to a particular class based on various features. The model is referred to as naïve since it makes the assumption that the input features are independent of one another.^42^

### Artificial Neural Network (ANN)

Artificial Neural Networks (ANNs) are computational models that are based on how the human brain is organized and functions. They are a subset of ML algorithms with the ability to identify patterns, anticipate outcomes, and perform various tasks. Neural network nodes, or artificial neurons, are the fundamental components of a neural network.^43,44^ Different layers, as shown in Figure 5 (input layer, hidden layers, and output layer), are used to arrange this node.

Input layer: Receives the input raw data. Hidden layers: Layers present in between input and output layers. Hidden layers use weighted connections to process the input data. Mainly two types of operations can be performed by hidden layers, i.e., weighted sum and activation of neuron. Output layer: Produces the final output or prediction. Neurons in one layer are fully connected to the neurons in the next layer. The information can be easily transmitted through these connections. Weights are assigned to each node, which determines the strength of the connection. These weights are easily adjusted to improve the network’s performance during model training. The different layers involved in ANN are shown in Figure 5G.

### Convolution Neural Network (CNN)

Convolutional Neural Networks (CNNs) are neural networks primarily used for analyzing visual images. CNN has the potential to automatically and adaptively learn spatial hierarchies of features, which makes them effective at picture recognition, object detection, and other visual tasks.^45,46^ Convolutional layer, pooling layer, and fully connected layers are the important layers present in the CNN model, as shown in Figure 5H. Convolutional layer: Multiple convolutional layers are present in CNN. At each layer, various filters (also called as kernels) applied to the input image to extract the important information. At convolution layer, filters are sliding over the input image produce feature maps. This mapping helps to highlight important features like edges, textures, or pattern in the image. Pooling layers: After the convolutional layers, pooling layers plays an important role and generally used to reduce the dimensionality of each feature map while retaining the most important information. Fully connected layers: The output of convolutional and pooling layers is flattened into single vector using flatten layer and then fed to the fully connected (dense) layers. The dense layer produces the final output of the CNN network, which may represent numerical values for regression tasks or probabilities across different classes for classification purposes.

Ultimately, this section gives readers a thorough grasp of a variety of ML models by explaining each one’s procedures and underlying ideas. A comparison of various models (Table 2) is shown in the following table, along with information on their benefits, drawbacks, and ways to improve comprehension. Readers will be better able to choose the best model for their particular requirements by learning more about these various algorithms, which will promote efficiency and innovation in the biosensor and predictive analytics industries. With this understanding, researchers and lawmakers may fully utilize ML, opening the door to breakthrough discoveries and predictions that are more accurate.

**Table 2:** Comparative Analysis for Different ML Models Used for Regression/Classification Problems^25,47−50^

**ML Model**	**Strength**	**Weakness**	**Applications**
Linear regression	Simple and easy to interpret	Assumes linear relationship, sensitive to outliers	Used when the relationship between features and target is approximately linear
	Fast training and prediction times		
	Works well with linear relationships between features and targets		
Random Sample Consensus	Robust to outliers in the data	May require a large number of iterations to converge	Computer vision, image stitching, feature matching. Used when dealing with data sets containing outliers and when robust estimation of parameters is desired
	Can handle noisy data sets effectively		
	Suitable for linear and nonlinear regression problems	Sensitivity to the choice of threshold parameters	
Theil-Sen Estimator	Robust to outliers and non-normality in the data	Computationally intensive for large data sets.	Used when robust estimation of parameters is critical and when dealing with data sets containing outliers
	Provides consistent estimates of parameters even with up to 29% of outliers	May not perform well with highly skewed data sets	
Decision Tree	Able to capture complex nonlinear relationships in the data	Prone to overfitting, unstable (small data changes can result in different trees)	Classification, regression, feature selection, decision analysis. Used when the relationship between features and target is nonlinear or when interpretability is important
	Easy to interpret and visualize		
	Robust to outliers in the data		
Random forest	Robust and less prone to overfitting compared to individual decision trees	Computationally intensive, less interpretable than a single decision tree	Classification, regression, feature selection, anomaly detection. Used when high predictive accuracy is desired and interpretability is less important
	Handles high-dimensional data sets with ease	Slower training and prediction times compared to decision trees Sensitive to the choice of kernel and hyper parameters	
	Provides feature importance scores		
Support Vector Machine	Effective in high-dimensional spaces		Classification, text categorization, image recognition. Used when dealing with nonlinear regression problems and when interpretability is less important
	Robust to overfitting, especially with appropriate kernel functions	Can be computationally intensive, especially with large data sets	
K-Nearest Neighbor	Simple and intuitive concept	Computationally expensive during prediction, especially with large data sets	Classification, regression, Pattern recognition, data imputation, anomaly detection
	No training phase, making it suitable for online learning	Sensitive to the choice of distance metric and number of neighbors	
	Effective for multiclass classification		
AdaBoost	It helps to reduces bias variance trade off, performs well with various weak learners	Sensitive to noisy data sets and outliers	Classification, regression, text recognition, face detection
Gradient Boosting	Provides high predictive accuracy, handles missing data very well, also reduces bias and variance trade off	It is prone to overfitting, computationally intensive	Classification, regression, ranking, anomaly detection
Extreme Gradient Boosting	High predictive accuracy and speed. Regularization techniques to prevent overfitting	Can be sensitive to noisy data	Regression, classification, anomaly detection pattern recognition Used when high predictive accuracy is desired and computational efficiency is important
	Handles missing data well	Requires tuning of hyper parameters	
Artificial Neural Networks	It is flexible and powerful models for a wide range of problems, robustness to noise, provides nonlinear modeling capability, provides parallel processing capabilities of GPUs	Prone to overfitting, requires large data sets and tuning	Regression, classification, anomaly detection pattern recognition
Long Short-Term Memory (LSTM)	Solves vanishing gradient problem, good for long sequence data	Computationally intensive, complex architecture	Time series forecasting, language modeling, speech synthesis
Generative Adversarial Networks (GAN)	Generates realistic data, unsupervised learning	Difficult to train, risk of mode collapse	Image generation, style transfer, data augmentation, creative applications
Bayesian Networks	Handles uncertainty, provides probabilistic interpretations	Computationally intensive, complex to implement	Diagnosis, forecasting, decision support systems

## Application of ML for PoCT

The incorporation of ML approaches has transformed biomarker detection across several modalities in the quickly changing field of PoCT. Calorimetry, electrochemiluminescence, electrochemistry, chemiluminescence, and microfluidics are examples of frontline techniques that provide unmatched biomarker detection sensitivity and specificity. This section explains how ML may be applied in a variety of ways to various methods, showing how they can work together to improve PoCT capabilities.

## ML Assisted Electrochemical Sensors for PoCT

These cutting-edge PoCT sensors, assisted by ML algorithms, provide unmatched precision as well as efficiency in identifying a wide range of biomarkers, revolutionizing medical diagnostics. Generally, electrochemical sensor detection methodology follows the following sequence for detection: 1) Chemical Interaction: Upon applying a sample to the sensor, specific chemicals inside the sample react with particular molecules on the surface of the sensor, 2) Electron Transfer: The electrical characteristics of the sensor are altered as a result of this interaction. In particular, it deals with the movement of electrons from the sample’s molecules to the sensor surface. 3) Signal Generation: A signal is produced electrically by the movement of electrons between the molecules. The signal’s magnitude corresponds to the target substance’s concentration in the specimen. 4) Measurement: The sensor then measures the electrical signal and transforms it into a readable output, like a color change or digital display. 5) Analysis: The sensor measures the concentration of the target material in the sample by evaluating the electrical signal’s strength.^51^ With the use of ML, electrochemical sensors can quickly process large, complicated data sets and identify minute patterns that may indicate a disease with remarkable sensitivity. This combination enables PoCT devices to provide accurate and timely data, accelerating the process of making decisions about diagnosis and therapy at the patient’s bedside. Numerous case studies show how this combination of ML with electrochemical sensing has been useful in a range of real-world scenarios. Following different case studies reveals how different groups used electrochemical sensors assisted with ML technology to detect various bioanalytes.

**Figure 6:**
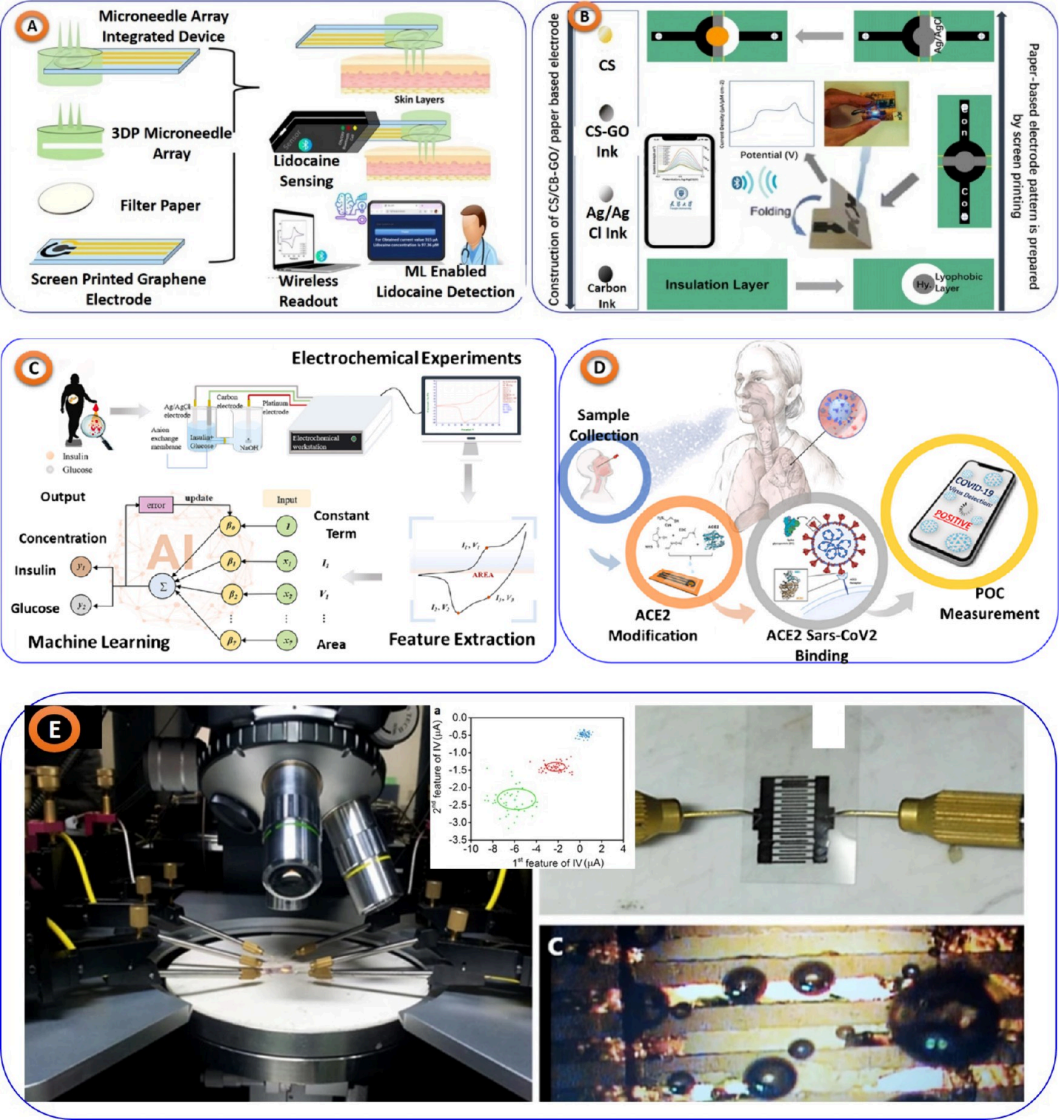
(A) Lidocaine detection using microneedle array integrated electrochemical PoCT sensor, taken from^52^ with the permission of Elsevier. (B) ML-assisted paper-based flexible electrochemical biosensor to detect tyrosine, taken from^53^ with the permission of Royal Society of Chemistry, Available under a CC-BY-NC license. (c) Insulin and glucose detection through ML in serum samples, replicated from^54^ with Elsevier permission. (D) laser-scribed graphene (LSG) sensors PoCT diagnosis of the SARSCoV-2 variant, taken from,^55^ with the permission Elsevier (Available under a CC-BY-NC-ND license). (E) Disposable biosensor for rapid detection and classification of bacteria, taken from,^56^ with the scientific reports permission (Available under a Creative Commons Attribution 4.0 International License).

The accuracy, sensitivity, and specificity of PoCT have been greatly improved by recent developments in ML-integrated electrochemical sensors. For instance, a lidocaine detection^52^ sensor using a wireless microneedle array demonstrated a detection limit of 0.13 μM, and ML algorithms accurately predicted the concentrations, (shown in Figure 6A). Similar to this, serum-based sensors, shown in Figure 6B, for glucose and insulin and paper-based biosensors for tyrosine detection, shown in Figure 6C, with ML assistance have demonstrated exceptional accuracy and speed, improving the effectiveness of diagnostic procedures.^53,54^ In addition, several case studies illustrate the diverse applications and benefits of ML-assisted electrochemical sensors in PoCT, from the detection of glucose, and SARS-CoV-2 variants^55^ (shown in Figure 6D). These sensors have demonstrated incredible sensitivity and accuracy, including over 99% accuracy in viral detection and quick diagnosis of numerous analytes in just a few minutes. Furthermore, innovations like bacterial detection^56^ (shown in Figure 6E), portable nitrate biosensors,^57^ models counteracting fouling effects^58^ and neurotransmitter identification^59^ highlight the broad potential and adaptability of these technologies in clinical settings.

The overarching trend in these studies demonstrates how integrating ML with electrochemical sensors significantly improves the accuracy, sensitivity, and specificity of PoCT devices. This synergy not only expedites the diagnostic process but also guarantees dependable, real-time data, crucial for immediate medical interventions. The ongoing developments in this area promise additional advances in personalized medicine by enabling a quick, accurate diagnosis customized to the specific requirements of each patient. Table 3 presents a different compilation of important works on ML assisted electrochemical sensors and their numerous potential uses. These tables showcase the wide range of research being done in this area and show how ML and electrochemical sensing technologies work well together in a variety of contexts.

**Table 3:** ML Assisted Electrochemical Sensors for PoCT Applications

**Sr. No**	**Sensor/Electrode Used**	**Applications**	**Regression/Classification ML Approach**	**ML Algorithms Used**	**Real Sample Analysis**	**ref**
**1**	Microneedle array with screen-printed electrodes	Lidocaine detection in artificial interstitial fluid (1–120 μM, LoD 0.13 μM)	Regression	Linear regression models	Microneedle array with screen-printed electrodes	(52)
**2**	Conventional three electrode system	Insulin and glucose	Regression	Linear regression	Serum	(54)
3	laser-scribed graphene (LSG) sensors	Coronavirus 2	Classification	Dense Neural Network	Swab samples	(55)
4	Printed electronic biosensor	*Salmonella typhimurium*, and the *Escherichia coli* strains JM109 and DH5-α	Classification	linear discriminant analysis, nonlinear back-propagation neural network	Pathogens samples	(56)
5	Screen-printed electrochemical sensors	Real-time monitoring of salt concentration (up to 10 mM), pH (4–10), and H_2_O_2_ (2 μM) in plant roots	Classification	eXtreme Gradient Boosting	Plant roots	(60)
6	Conventional three electrode system	Nitrate determination	Regression	SVM	lake water, vegetable juice and fruit juice	(57)
7	Conventional three electrode system	Propofol	Classification	Support vector classifier	Human serum	(58)
8	Graphene-Modified Carbon Electrode	Dopamine and Serotonin	Regression	ANN	-	(59)
9	Two gold electrodes on polyimide substrate	Cortisol (8–140 ng/mL)	Classification	kNN	Sweat	(61)
10	Disposable laser-induced porous graphene (LIPG) electrode	Maleic hydrazide (MH) detection in potatoes and peanuts (0.9–101.9 mM)	Regression	ANN, RF and SVR	Real sample analysis was performed using potatoes and peanuts	(62)
11	Microbial electrochemical sensors (MECs)	Quantification of multiple toxicants in water	Regression	SVM, ANN,	Microbial electrochemical sensors (MECs)	(63)
12	Electrodeposited molybdenum polysulfide (eMoSx) on Laser-induced graphene (LIG)	Multiplexed detection of tyrosine (TYR) and uric acid (UA) in sweat and saliva (LODs of 100 nM for TYR and 10 nM for UA)	Regression	kNN, and DT	Electrodeposited molybdenum polysulfide (eMoSx) on Laser-induced graphene (LIG)	(64)
12	TiO_2_ nanotubes	Hydrogen sensing (0.5–10% H_2_concentration)	Classification	SVM, and ANN	TiO_2_ nanotubes	(65)
14	Modified electrode with silver nanoparticles	Rapid detection of Sudan Red I in food (0.1–20 μM, LoD 10 nM)	Regression	CNNs	Food samples	(66)
				Inception V1 and ResNet-50		
15	CuO/rGO/NPAN composite electrodes	Carbendazim residue detection in tea (0.5–100 μM, LoD 0.19 μM)	Classification	SVM, RF	Tea samples	(67)
16	Microfluidic chips composed of a single piece of PDMS	Metal ions	Classification	linear discriminant analysis, partial least-squares and RF	Lake samples	(68)
17	Conventional three electrode system	Glucose	Regression	SVM, ANN	-	(69)

## ML Assisted Colorimetric Sensors for PoCT

Colorimetric sensors, aided by ML, are gaining importance in PoCT applications as they enable sensitive, selective, and rapid detection of various analytes with minimal equipment and expertise required. Here’s how colorimetric sensors work: 1) Chemical Reaction: Target analyte and reagent on the sensor surface undergo particular chemical reactions when a sample is added to the sensor. 2) Color Change: As a result of this chemical reaction leading to a change in color, which is directly proportional to the concentration of the target bioanalytes present in the sample. 3) Image Capture: The color change can be captured using different imaging mechanisms. 4) Data Processing: Based on the different image processing softwares or using ML models, the concentration of bioanalytes is calculated/predicted. The following case studies demonstrate how ML-assisted colorimetric sensors identify a variety of bioanalytes, redefining patient care by increasing specificity, sensitivity, and accuracy while cutting down on expenses and time spent on diagnosis.

**Figure 7:**
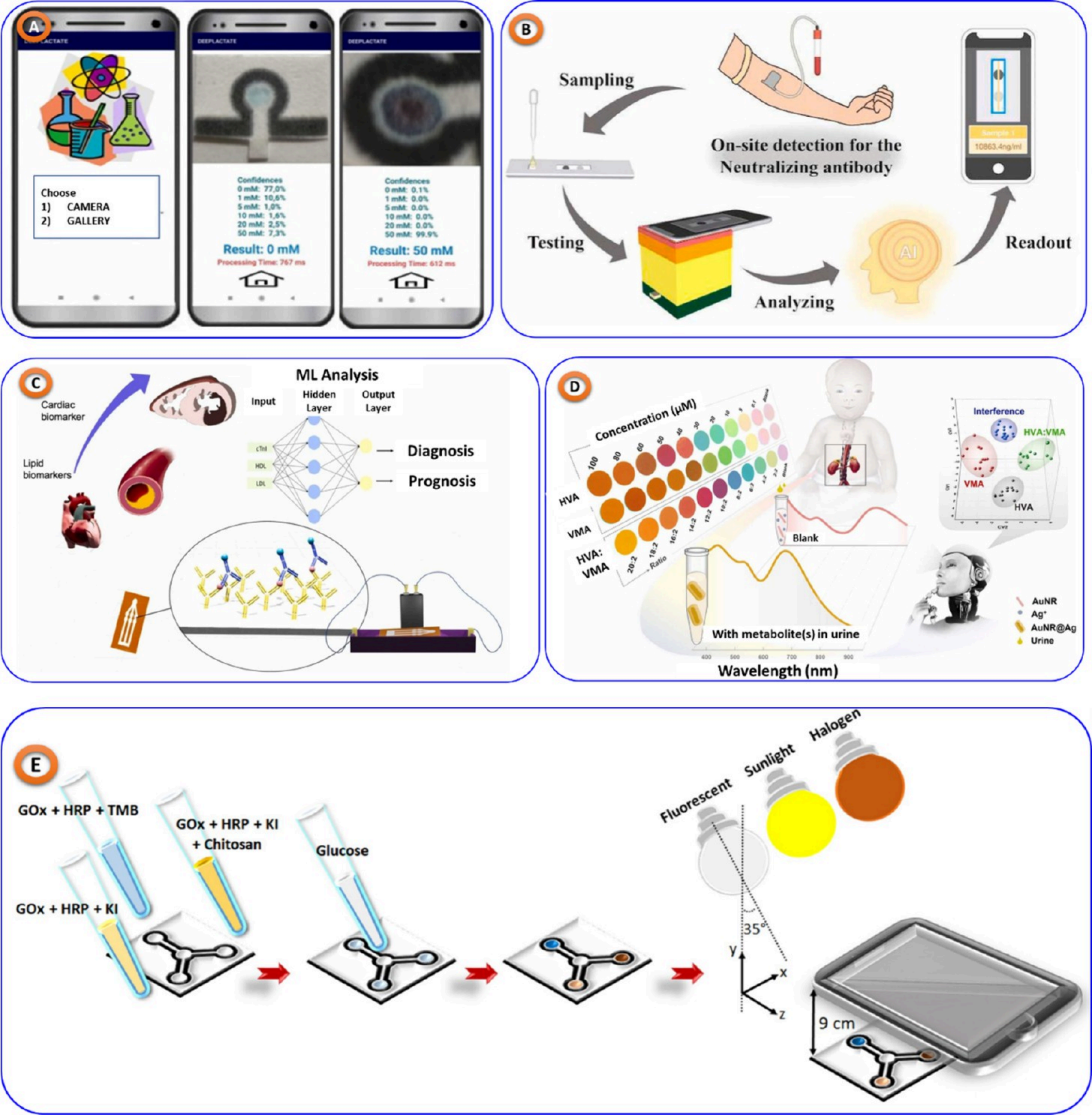
(A) Microfluidic paper-based analytical device to detect lactate in sweat, replicated from,^70^ copyright Elsviwer. (B) deep learning assisted colorimetric sensor for neutralizing antibodies postvaccination detection, taken from,^73^ copyright Elsviwer. (C) Paper based microfluidic colorimetric biosensor swift disease diagnosis and prognosis, taken from,^75^ copyright Elsviwer. (D) AI-enabled multicolorimetric sensor array for neuroblastoma urinary marker detection, taken from,^76^ copyright Elsviwer. (E) Smartphone-coupled μPAD system employing ML to detect glucose in artificial saliva, replicated from,^72^ copyright Elsviwer.

For example, Microfluidic paper-based devices with great accuracy and cost-effectiveness have been developed for lactate^70^ (Figure 7A), pH, and glucose^71,72^ (Figure 7E) monitoring. AI-assisted immunoassays,^73^ shown in Figure 7B, have improved the evaluation of neutralizing antibodies postvaccination, while smartphone-assisted sensors have enabled inexpensive environmental monitoring for pollutants like lead.^74^ Additionally, innovative biosensors have been created for detecting cardiac biomarkers^75^ (shown in Figure 7C) and neuroblastoma^76^ (shown in Figure 7D) markers, providing rapid and precise diagnostic information.

These developments highlight the exciting possibilities of merging colorimetric sensing with ML in a variety of applications, from environmental monitoring to healthcare, and they additionally point to a larger trend of increased specificity, sensitivity, and accessibility in diagnostics. This holistic approach not only accelerates diagnosis but also enhances the reliability and ease of use of PoCT devices, ultimately improving patient care and treatment outcomes. Apart from these above-mentioned case studies, Table 4 offers a thorough summary of the many ML-assisted colorimetric sensors and their many applications, demonstrating the in-depth study and effective integration of these technologies in several domains.

**Table 4:** ML Assisted Colorimetric Sensors for PoCT Applications

**Sr. No**	**Sensor/Electrode Used**	**Applications**	**Regression/Classification ML Approach**	**ML Algorithms Used**	**Real Sample Analysis**	**ref**
1	Multicolorimetric sensor array	Homovanillic acid (HVA) and Vanillylmandelic acid (VMA) detection	Classification	Linear discriminant analysis and Principal component analysis	Urine	(76)
2	μPADs (Paper-based sensors)	Glucose	Classification	Linear discriminant analysis		(77)
				Gradient Boosting Classifier and RF		
3	Paper-based sensors	H_2_O_2_	Classification	Linear Discriminant Analysis	Tap water and artificial serum	(78)
4	Gold nanoparticles (Au NPs) colorimetric sensors	Glutathione (GSH)	Regression	ANN	-	(79)
5	High- throughput colorimetric sensors (c-sensor)	Total organic carbon (TOC)	Regression	CNN based on LeNet-5	Environmental water samples	(80)
6	Colorimetric sensor array using oxidized chitin nanocrystals (O-ChNCs)	Monitoring beef freshness	Classification	CNN	Beef samples	(81)
		Methylamine (MA): 100 ppm				
		Trimethylamine (TMA):70 ppm				
		Ammonia (NH3): 70 ppm				
7	Colorimetric strips	Sulfate, Ammonium, Arsenic, Nitrate	Classification	kNN	Local water sources	(82)
8	Microfluidic paper-based analytical device (μPAD)	Lactate (0.67 mM LoD)	Classification	Inception-v3 (CNN)	sweat	(70)
9	Colorimetric polydopamine nanoparticle (PDA)-based lateral flow immunoassay (LFIA)	COVID-19 neutralizing antibody quantification (625–10000 ng/mL, 160 ng/mL LoD)	Regression	vision transformer	serum	(73)
10	Smartphone-based colorimetric sensor using gold nanoparticles	Lead detection in water (0.5–2000 ppb, 0.5 ppb LoD)	Regression	Modified ReLU	Water Samples	(74)
11	Deep learning based Mask R-CNN model (Image Processing)	Nucleic acid quantification in microarray and droplet dPCR	Classification	Mask R-CNN	Digital PCR fluorescence images	(83)
12	Colorimetric paper sensor	pH and glucose detection (pH 2–10, 0–10 mg/mL glucose) pen_spark	Regression	RF achieved highest accuracy	Serum	(84)
13	Hand-held smartphone-based colorimetric microplate reader (micro plate)	Cancer cell line detection	Regression	AdaBoost	Cell lines and cell culture	(71)
14	Paper-based analytical device	Cardiac troponin and lipid biomarker quantification	Classification	CatBoost	Serum	(75)

## ML Assisted Lab-on-Chip Sensors for PoCT

By enabling intricate biochemical reactions and analysis on a single chip, microfluidic technologies greatly minimize the amount of reagents and sample sizes needed. ML assisted lab-on-a-chip sensors provide high-throughput screening capabilities and automated data interpretation by integrating complex algorithms with microfluidic technology. This integration not only enhances patient outcomes but also aids the larger healthcare system by cutting overall healthcare expenditures and the need for substantial laboratory infrastructure. Mainly following steps are involved while performing sensing using Lab-on-Chip devices. 1) Sample Collection and Preparation: Small amounts of biological samples (in μL), such as blood, saliva, and urine, are collected and transported through microfluidic channels. 2) Microfluidic Manipulation: In order to route samples to different sensing locations, the microfluidic system regulates the flow of fluids through channels, valves, and pumps. 3) Chemical and Biological Reactions: In specific regions of the chip, particular biochemical events take place. These reactions frequently involve the interaction of target analytes with antibodies, enzymes, or nucleic acids. 4) Signal Detection: Integrated sensors track the chemical or physical alterations brought about by the reactions. These modifications are converted to electronic signals by mechanical, optical, or electrochemical sensors. On the other hand, this method not only expedites the diagnosis process but also reduces healthcare expenses, increasing accessibility to high-quality diagnostics. Lab-on-a-chip sensors with ML support are changing patient care by enabling prompt and efficient medical interventions. This section delves how Lab-on-Chip devices with ML is used for diagnosis purpose.^85,86^

**Figure 8:**
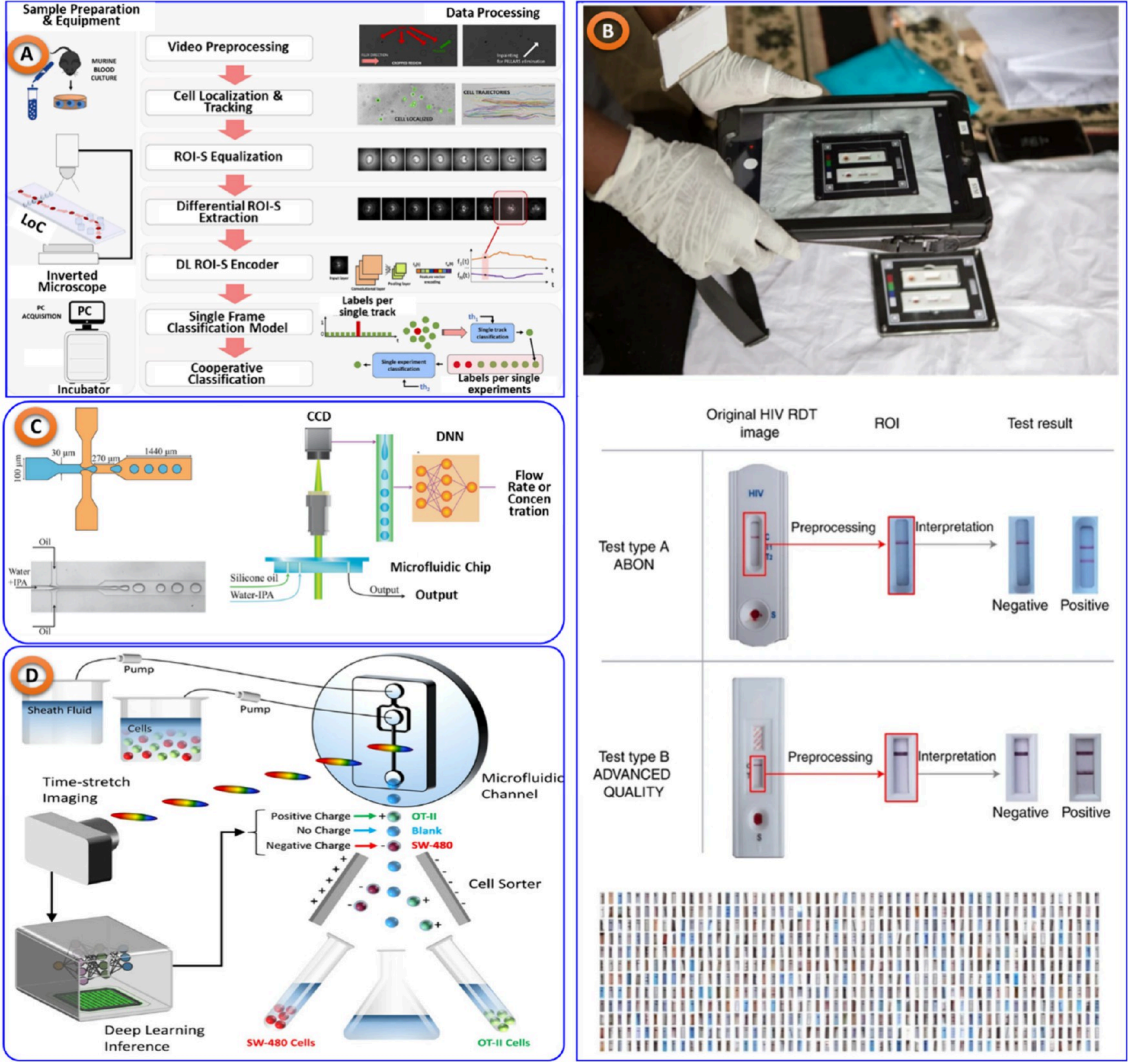
(A) Microfluidic technology to detect murine red blood cell samples in blood diseases, taken from^87^ with Elsevier permission. (B) Deep learning assisted microfluidic device to perform HIV tests, recreated from^88^ with Springer Nature permission. (C) Deep Deep learning assisted microfluidic chip to measure flow rate, taken from^89^ with Springer Nature permission, Available under Creative Commons CC BY license. (D) ML assisted microfluidic system to classify cancerous cell,^90^ with the permission of science report, Available under Creative Commons CC BY license.

First, Mencattini et al.^87^ applied deep learning models with microfluidics devices (shown in Figure 8A) to detect murine red blood cells in blood diseases leads to achieve over 85% accuracy, which ultimately improves the real-time diagnostic capabilities. Another study proposed by Valérian Turbé et al.^88^ uses of deep learning to analyze HIV test images and achieved with 97.8% sensitivity and 100% specificity, increasing the speed and accuracy of HIV diagnosis, shown in Figure 8B. Pooria Hadikhani et al. suggested an optical technique that measures (shown in Figure 8C) microfluidic droplet flow using neural networks to get accurate measurements of the flow rate and concentration, thereby enhancing diagnostic efficiency.^89^ With the use of deep learning, Yueqin Li and colleagues^90^ were able to classify cells in microfluidics system (shown in Figure 8D) without labels, detecting white blood cells and cancer with over 95% accuracy, promising improved early cancer detection and efficient cell sorting.

Together, these advancements improve diagnostic precision, reduce costs, and facilitate quick, trustworthy medical testing. By enabling easier and faster access to diagnostics, these advances have the potential to change patient care. Furthermore, Table 5 provides an extensive overview of ML-assisted Lab-on-Chip sensors and their many uses, demonstrating the thorough investigation and effective integration of these technologies across numerous domains.

**Table 5:** ML Assisted Lab-on-Chip Sensors for PoCT Applications

**Sr No.**	**Application**	**Regression/Classification ML Approach**	**ML Algorithms**	**Real Sample Analysis**	**ref**
1	Red blood cell plasticity evaluation (Kinase Disease monitoring)	Classification	Deep Learning (AlexNEt, ResNet101, NasNetLarge)	Blood	(87)
2	Detection of Giardia cyst and Cryptosporidium cyst in food and water	Classification	CNN	Blood, Tissue, Parasites	(91)
3	Malaria, HIV, syphilis, tuberculosis, influenza and noncommunicable diseases	Classification	SVM,CNNs- ResNet50, MobileNetV2 and MobileNetV3	Blood	(88)
4	flow rate of water/alcohol mixture	Classification	Deep neural networks	Water	(89)
5	Detection of Cancer Cell in blood	Classification	Deep neural network, Convolution, ReLu, CNN	-	(92)
6	Cancer screening	Classification	CNN GoogLeNet42, ResNet1831, AlexNet43, SqueezeNet44, and Inceptionv3	Tissue	(93)
7	Counts RBC,WBC, and platelets, anemia, to monitor the progression of HIV/AIDS	Regression	Extreme Learning and CNN	Blood cell and HepG2 tumor cell	(94)
8	Identification of tumor or metastasis	Classification	NB, RF tree, LR, kNN, stochastic gradient descent, neural network and Adaboost	Tumor Biopsy	(95)
9	Contact-Imaging Based Microfluidic Cytometer	Regression/Classification	Extreme Learning Machine (ELM) &SR	Cell	(96)

## ML Assisted Electrochemiluminescence/Chemiluminescence Systems for PoCT

In order to measure the presence of particular biomarkers, electrochemiluminescence (ECL) and chemiluminescence (CL) sensing techniques in PoCT devices use light emitted from chemical reactions. In the case of CL systems, a patient sample is mixed with reagents (e.g., Luminol, H_2_O_2_ and Cobalt) that react with the target molecules, producing light proportional to the amount of these molecules. Be specifically, to produce light no external power supply is needed to initiate reaction.^97,98^ On the other hand, ECL systems function similarly, but they also require the application of an electric current to initiate a chemical reaction.^99,100^ This improves the detection process’s sensitivity and specificity. Both approaches yield quick and precise findings, which make them perfect for PoCT devices that may be utilized in a variety of contexts, such as clinics or remote areas, and require little user training. ML assisted ECL and chemiluminescence CL systems play an important role in PoCT by improving the accuracy and efficiency of diagnostic procedures. These ECL/CL systems use ML algorithms to interpret large, complicated data sets, reduce reaction times, optimize assay conditions, and improve signal detection. This improves test results’ sensitivity and specificity. Following are several studies highlighting the innovative applications of ECL and CL systems.

**Figure 9:**
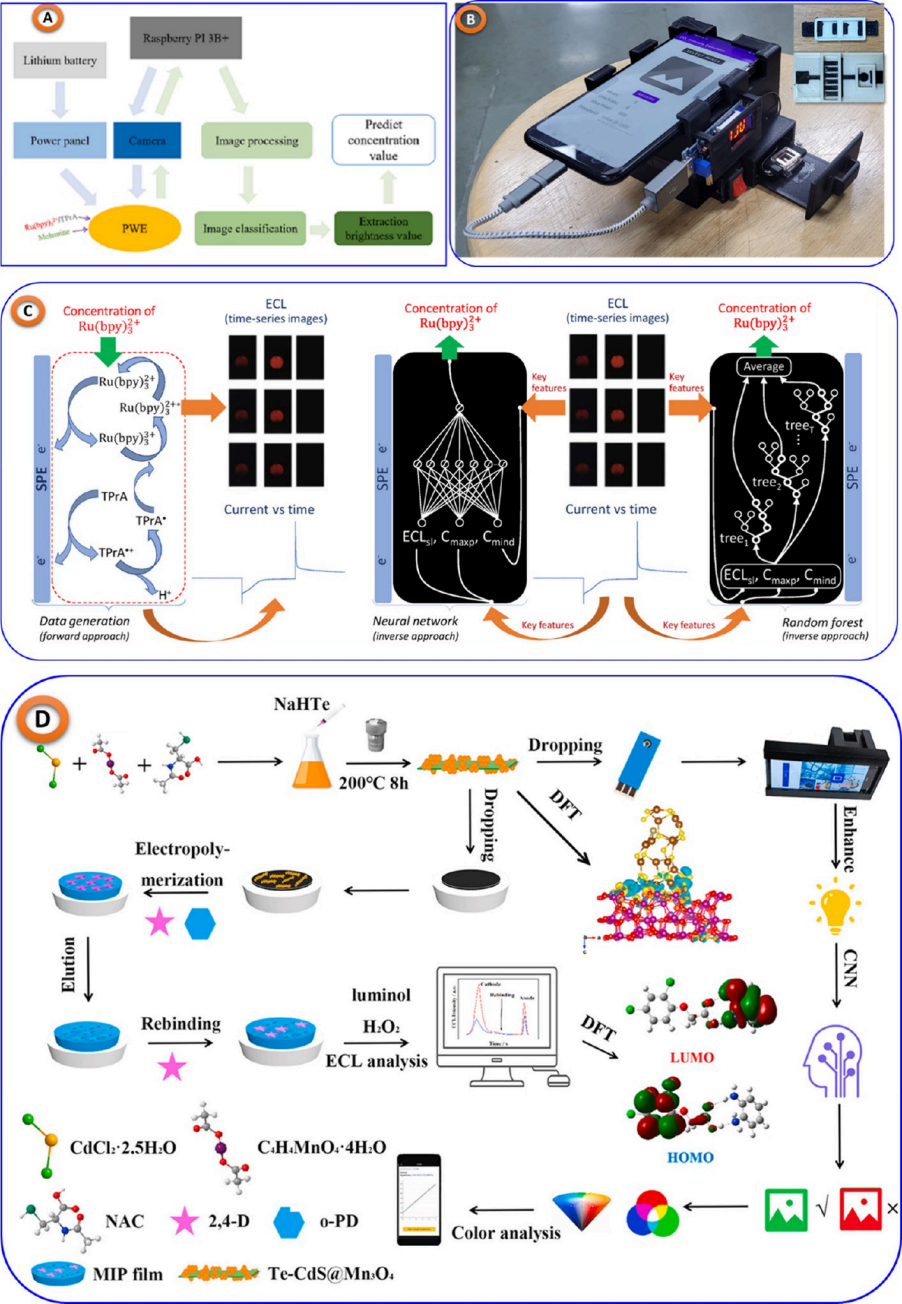
(A) Portable miniaturized ECL imaging system leveraging Raspberry Pi technology for real time detection of melamine, replicated from,^101^ copyright Elsevier. (B) 3DP black box Assisted with mobile phone for various bioanalytes detection, taken from^108^ with the permission of IEEE. (C) Mobile phone assisted ECL imaging system for ruthenium detection, taken from,^104^ copyright MDPI. (D) ML-assisted smartphone-based platform to selectively detect 2,4-Dichlorophenoxyacetic acid, taken from,^105^ with the permission of Elsevier.

**Figure 10:**
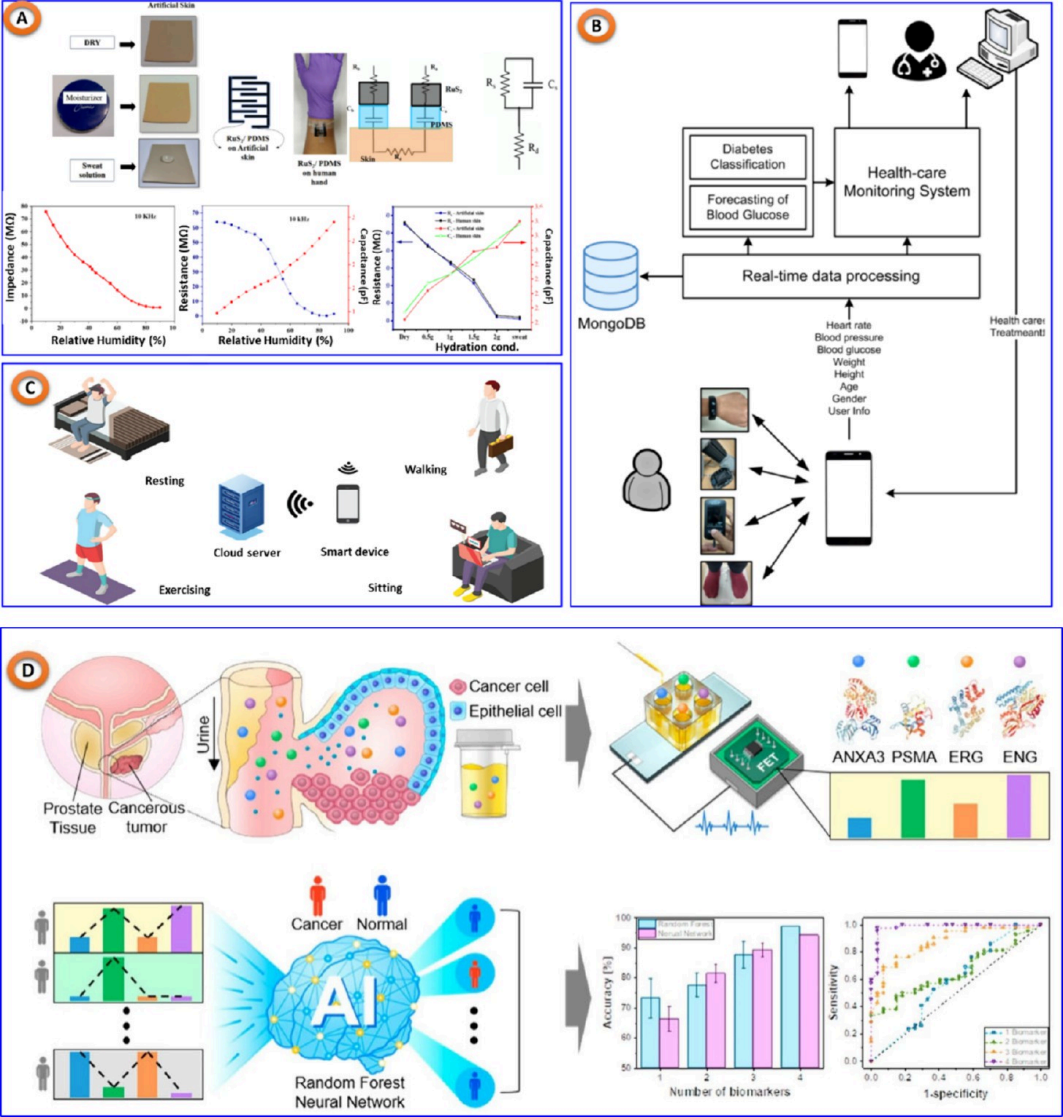
(A) AI/ML assisted multifunctional sensing platform for simultaneous detection of glucose, and pH in sweat, reproduced from,^119^ copyright MDPI. (B) Personalized healthcare monitoring system, taken from.^120^ (C) A ML assisted sweat glucose reporting platform, replicated from,^122^ copyright MDPI. (D) Noninvasive precision screening of prostate cancer: ML approach, replicated from,^125^ with the permission of American Chemical Society.

For example, Yipeng Li et al. developed a portable AI-ML assisted ECL imaging system^101^ for melamine detection, shown in Figure 9A, achieving a detection limit of 3.54 nM. Manish Bhaiyya and team proposed a miniaturized 3D-printed ECL platform for cholesterol,^102^ glucose, lactate,^103^ and choline^104^ detection using smartphones and ML models, enhancing diagnostic accuracy (shown in Figure. 10B). Elmer Ccopa Rivera et al.^104^ applied AI with ECL sensors for rapid substance identification, shown in Figure 9C. In another study, Zhiwei Lu’s et al.^105^ developed a smartphone-based ECL platform for detecting 2,4-Dichlorophenoxyacetic acid, shown in Figure. 10D. Additionally, Anatoliy Kazak’s^106^ group used CL methods for assessing antioxidant activity in wines, and Ali A. Ensafi et al.^107^ developed a CL method for detecting noscapine and thebaine.

The presented methodology showed high accuracy and low detection limits, indicating its potential for pharmaceutical analysis and clinical diagnostics. These sensing innovations combined with ML collectively enhance diagnostic accuracy, sensitivity, and specificity while reducing costs and time, making advanced diagnostics more accessible and efficient. The various methodologies, target analytes, detection limits, ML techniques, and specialized applications are clearly summarized in this comparison Table 6, which highlights the efficacy and adaptability of ECL and CL systems in PoCT.

**Table 6:** ML Assisted ECL and CL Systems for PoCT

**Sr No.**	**Sensing Approach**	**Fabrication Method**	**Application**	**Regression/Classification ML Approach**	**ML Algorithm Used**	**Real Sample Analysis**	**ref**
1	ECL	Disposable screen-printed carbon electrodes	Ruthenium (0.02 μM –2.5 μM)	Regression	RF and Feed forward network	-	(104)
2	ECL	3DP Electrodes	Glucose (0.05–3 mM; 0.033 mM)	Regression	LR, DT, RF, SVR	Blood Serum	(102)
			Choline (0.1–4 mM; 0.07 mM)				
			Lactate (0.0007–1 mM; 0.0007 mM)				
3	ECL	Screen-printed electrodes based MIRECL sensor	2,4-Dichlorophenoxyacetic acid(2,4-D)	Classification	CNN (100)	Soil, Water, Pomelo peel, Mango peel	(105)
4	ECL	3DP Electrodes	Glucose (0.1 mM to 1 mM; 0.04 mM)	Regression	For Glucose/lactate	Blood Serum	(103)
			Lactate (0.1 mM to 4 mM; 0.1 mM)		• RF (0.97/0.99)		
					• LR (0.92/0.91)		
					• RF (0.97/0.99)		
					• KNN (0.98/0.99)		
					• AdaBoost (0.98/0.98)		
5	ECL	3D printed graphene filament based closed bipolar electrode ECL sensor	Glucose (0.5 to 10 mM; 0.49 mM), Lactate (0.01 to 1 mM; 0.01 mM), Choline (0.1 to 5 mM; 0.09 mM)	Regression	For Glucose/Lactate/Choline/Cholesterol	Blood serum	(108)
			Cholesterol (0.5 to 10 mM; 0.3 mM)		• Huber (0.96/0.95/0.94/0.97)		
					• RANSAC (0.96/0.92/0.95/0.97)		
					• Theil-Sen (0.97/0.92/0.94/0.9)		
6	ECL	Molecularly imprinted polymer (MIP) ECL sensors	Furosemide (1 μM to 70 μM; 0.25 μM)	Classification	CNN (91.5)	Human urine and pill samples	(109)
7	CL	-	Antioxidant Activity	Regression	LR, neural network regression model	Wine	(106)
8	CL	-	Thebaine and noscapine	Regression	SVR	Human plasma	(107)
9	ECL	Synthesized nanomaterials were used	Dopamine (0.1 nM to 1 mM)	Regression	Deep neural network	Serum	(110)
10	ECL	Paper based ECL device	Coronavirus 2 (SARS-CoV-2)	Regression	ANN	Artificial samples.	(111)

## ML Assisted Wearable Sensors for PoCT

Wearable sensors can be used to continually monitor a variety of physiological characteristics by being directly attached to any area of the body or incorporated into accessories like watches, glasses, necklaces, and bracelets.^112^ ML assisted wearable sensors for PoCT, which combine wearable technology with ML to monitor and analyze health data in real time, represent significant development in healthcare technology. Among the many benefits these sensors provide is the ability to monitor vital signs in real-time, such as blood pressure, glucose levels, and heart rate, which makes it easier to identify possible health problems early on.^113,114^ By spotting anomalous trends in the data, ML algorithms can forecast health issues before they get out of hand. Personalized healthcare is another benefit of this technology, which offers specialized medical advice and treatments based on patient information.^115^ There are multiple processes involved in using these wearable sensors. First, they use a variety of embedded sensors to gather physiological data; for instance, let us assume biosensors to measure glucose levels, photoplethysmography (PPG) sensors to measure heart rate, and accelerometers to measure activity. After that, the data are wirelessly transferred, often via Bluetooth to a cloud-based system or a smartphone for processing. ML algorithms to find patterns, anomalies, and trends process the data. These findings are then used to give the user feedback in the form of advice, alerts, and health-related information.^116,117^ Following the discussion of the benefits and features of ML assisted wearable sensors for PoCT, it is critical to investigate practical uses and the effects these technologies have had in a range of healthcare settings. The following case studies demonstrate how these cutting-edge wearable sensors have been effectively used to enhance personalized healthcare, provide early disease detection, and improve patient outcomes.

In that sense, Veeralingam and colleagues^118^ developed an AI/ML-assisted multifunctional sensing platform to detect sweat pH levels, glucose levels, and skin hydration, shown in Figure 10A. The platform interfaces with a microcontroller board to analyze data using the KNN approach. Bogue-Jimenez’s group^119^ investigated the use of different ML algorithms in noninvasive continuous glucose monitoring forecast blood glucose levels based on biometric information such as body temperature and heart rate. Similar to this, Alfian’s team^120^ suggested a customized healthcare monitoring system (shown in Figure 10B) to manage chronic illnesses and anticipate diabetes utilizing real-time machine learning and BLE-based sensor devices. Furthermore, Kumari et al.^121^ proposed a continuous blood glucose measurement (CBGM) created a data-driven method integrating customized calibration and ML. Another important method proposed by Devangsingh Sankhala and group,^122^ where they have proposed a novel noninvasive sweat sensor technology that uses affinity capture probes and electrochemical impedance spectroscopy to quantify the amounts of glucose in human eccrine sweat, shown in Figure 10C. ML algorithms are then used to translate the samples into continuous glucose readings.

After talking about the capabilities and advantages of wearable sensors with ML assistance for PoCT, it is important to look into the real-world applications and impacts these technologies have had in different healthcare contexts. These state-of-the-art sensors improve patient outcomes, enable early disease identification, and promote a tailored treatment. They can be used to track vital signs, including heart rate, blood pressure, and glucose levels, in real-time, which enables the early detection of possible health issues. By identifying unusual trends in the data, AI and ML algorithms can forecast health problems. Furthermore, a number of applications including lung cancer diagnosis,^123^ COVID-19 detection,^124^ prostate cancer screening (shown in Figure 10D),^125^ and more have demonstrated the widespread applicability and impact of ML assisted systems in contemporary healthcare.

## ML Assisted Electronic-Nose (E-Nose) for PoCT

**Figure 11:**
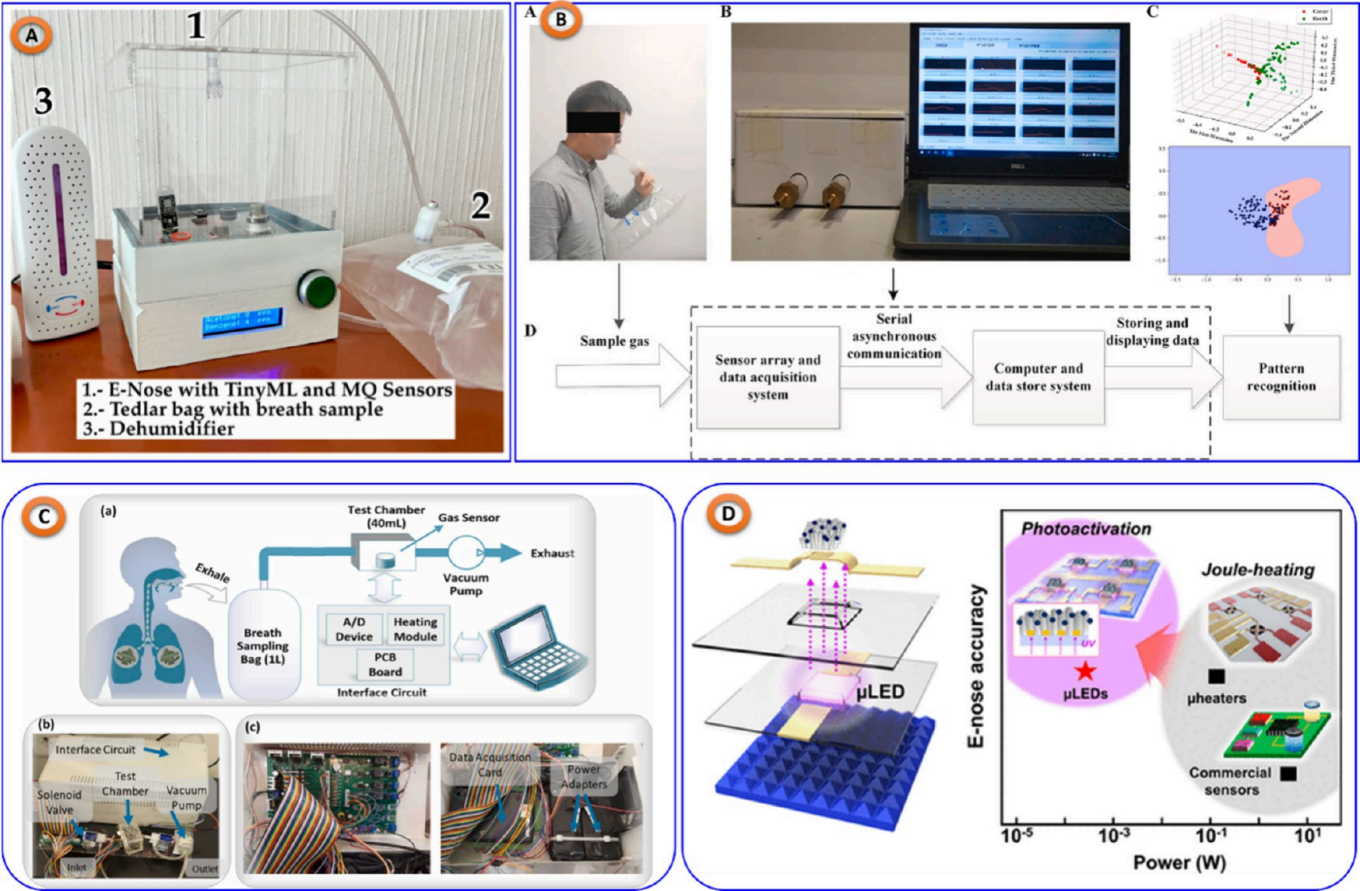
(A) E-Nose for diabetes detestation, replicated from,^128^ copyright MDPI. (B) E-Nose for lungs lung cancer and stages classification, taken from,^129^ with the permission of Elsevier. (C) E Nose for Chronic obstructive pulmonary disease detection, taken from,^130^ copyright Elsevier. (D) E-Nose for different gas sensing applications, taken from,^134^ copyright ACS.

The E-Nose operates by the use of a variety of gas sensors, each of which is sensitive to a different volatile organic compound (VOC). Based on the chemical composition of the VOCs present, these sensors generate distinct electrical signals when they come into contact with a sample. After processing the data, important characteristics are taken out to create a unique “odor fingerprint.” These fingerprints are analyzed using ML methods, to find patterns that are associated with specific illnesses or ailments.^126,127^ The E-Nose’s diagnostic capabilities are greatly improved by this ML integration, enabling the quick and precise diagnosis of conditions including diabetes^128^ (shown in Figure 11A), respiratory infections such as lung cancer^129^ and Chronic obstructive pulmonary disease^130^ (shown in Figure 11B,C), food quality control (Tea,^131^ Beer,^132^ Fraudulent Rice^133^), Gas sensing, (shown in Figure 11D)^134^ (air, ethanol, NO_2_, acetone, methanol) and other metabolic illnesses.^135,136^ The noninvasive nature of this technology, coupled with its ability to provide real-time results, makes it particularly valuable for PoCT applications

## Operational Challenges and Solutions in Clinical Settings

Through a comprehensive review of the literature, it is evident that the integration of ML in PoCT devices has ushered in a new era of smarter, more efficient, and accurate on-site healthcare diagnostics. Technologies such as electrochemical, colorimetric, ECL, lab-on-a-chip, and wearable sensors are crucial components of PoCT, each with unique diagnostic capabilities. Leveraging these advanced diagnostic technologies with ML enables more precise handling of complicated data sets as well as facilitates real-time decision-making. Despite these advantageous features, significant challenges must be overcome to realize their commercialization potential. These significant challenges are broadly summarized and outlined as follows.^137,138^

### Quantification and Analysis

The accurate measurement of signals generated by PoCT devices is a crucial component of their functionality. These signals may be optical, integrated, mechanical, or electrical in nature and must be quantified with precision to provide actionable data for medical and environmental monitoring purposes. ML algorithms are increasingly being utilized to enhance the performance of these devices, ensuring that the data they produce are reliable, precise, and effective in guiding decision-making. Despite the significant benefits offered by ML in this context, several challenges must be overcome to achieve the optimal results. These challenges include issues of sensitivity, selectivity, stability, repeatability, integration, data processing, and accessibility. To address these challenges, innovative solutions are required that take advantage of ML’s capabilities. In terms of sensitivity and selectivity, ML algorithms can help optimize the use of nanomaterials, bioreceptors, and signal amplification methodologies to achieve more accurate and reliable results. Additionally, ML can improve the stability and repeatability of PoCT devices by compensating for environmental fluctuations and ensuring a consistent data output over time. Furthermore, ML can enable the integration of multiple sensor functions on a single lab-on-a-chip platform, thereby enhancing the overall system efficiency. To ensure that data analysis and interpretation are performed effectively, ML and cloud-computer-based methodologies are essential. These tools enable the real-time analysis of complex sensor data sets, providing rapid and accurate diagnostic information. Finally, ML can enable the scalability of PoCT device manufacturing and ensure accessibility for a wide range of users by providing user-friendly interfaces and handling information.^139,140^

### The Transformative Role of ML in PoCT Biosensors

The utilization of ML technologies has a profound and transformative effect on PoCT biosensors, particularly in enhancing their accuracy, efficiency, and accessibility in clinical settings. These advancements have completely revolutionized data collection and interpretation, successfully overcoming the limitations of manual processes. Through the integration of ML with biosensors, healthcare outcomes have been greatly improved by providing more precise diagnostics and personalized treatments. In this article, we discuss the importance, advancements, challenges, and future prospects of ML in clinical applications via biosensors.^141,142^

ML integration in PoCT biosensors has drastically enhanced data analysis, predictive analytics, and tailored healthcare, resulting in early diagnosis and more personalized treatments. With the capability to process large amounts of complex data with high precision and efficacy, ML technologies significantly improve clinical evaluations. However, ML still faces challenges such as data uncertainty and limited labeled data. To address these issues, data supplementation, collaborative data sharing, and artificial data generation are some strategies that can be employed to create high-quality and diversified data sets, improving ML performance in clinical settings. The complexity of AI models poses another challenge to their interpretation. However, basic models, explainable AI (XAI), and posthoc analysis tools can be utilized to make ML models more transparent and understandable, increasing clinician trust and usability. Furthermore, seamless data integration and compatibility with existing clinical systems are crucial to effective implementation. Standardized protocols, gateway technologies, and cross-disciplinary collaboration can mitigate these challenges, enhancing the functionality of the ML-powered biosensors.

ML has immense potential for future applications in PoCT biosensors, such as automated validation and maintenance of devices, advanced statistical analysis for early disease diagnosis, and improved precision and sensitivity for detecting complex biomarkers. ML will also facilitate global data communication, driving innovation and improving healthcare outcomes.

IoT enables real-time data communication across PoCT devices, allowing for continuous health monitoring, tracking, and remote access. This results in immediate feedback, prompt interventions, and automated data collection, reducing the workload on healthcare providers and patients. Developing standardized protocols for device connectivity and ensuring data security and privacy are primary challenges that can be addressed through encryption, access controls, and secure communication protocols.

Cloud-based systems offer flexible and scalable data management, enabling continuous, real-time data collection and analysis. They facilitate global access and collaboration among healthcare professionals and researchers, improving diagnostic accuracy, and promoting personalized medicine. Cloud-based solutions support advanced analytics and AI integration, allowing for accurate forecasting and preventive healthcare management. This enhances remote monitoring and telemedicine capabilities, leading to better patient outcomes through continuous monitoring and early interventions. Maintaining data security and privacy, ensuring smooth integration and connectivity, and processing large amounts of data in real-time are key challenges that can be addressed through encryption, adherence to regulatory standards, and secure interoperability via standardized protocols, APIs, and edge computing.

### Regulatory Compliance and Clinical Validation

The integration of ML assisted PoCT offers significant developments in diagnostics and patient care. However, these technologies must undergo comprehensive regulatory and clinical validation to verify their safety, efficacy, stability, and reliability. This discussion covers key regulatory frameworks, including FDA approvals, CE markings, and other relevant certifications, highlighting the critical importance of adherence to clinical validation protocols.

The U.S. Food and Drug Administration (FDA) plays a pivotal role in regulating medical devices, including AI-ML assisted PoCT. The FDA categorizes medical devices into three classes based on risk: Class I (low risk, e.g., hand-held surgical instruments), Class II (moderate risk, e.g., virto test kits), and Class III (high risk, e.g., implantable devices). ML assisted PoCT devices are often classified as Class II or III due to their direct impact on the diagnosis and treatment of patients. Based on the classification, the approval process may vary and include 4 major key steps. Presubmission involves prior interaction with the FDA to establish the regulatory pathway. Devices substantially equivalent to an already marketed device require 510 (k) clearance. De Novo Classification is required for innovative devices that do not have an established criterion. High-risk devices must obtain Pre-Market Approval (PMA), which necessitates considerable clinical data. This process includes thorough clinical studies to demonstrate both safety and effectiveness, adherence to Quality System Regulation (QSR), which includes appropriate manufacturing standards, and postmarket surveillance for continued monitoring and reporting of device performance.^143^

In the European Union, the CE mark represents compliance with health, safety, and environmental protection regulations for devices sold inside the European Economic Area (EEA). The approval procedure starts with classification, which determines the medical device’s risk class (I, II, and III). The conformity evaluation varies by the device class, application, and technology. Technical documentation must fully demonstrate conformity with EU rules, and clinical data are evaluated to establish device performance and safety. Key factors include conformity to the Medical Device Regulation (MDR), which assures severe evaluation processes, and postmarket surveillance, which entails continual monitoring to ensure continuing compliance

Other relevant certifications include Health Canada Approval, which incorporates identical procedures to the FDA and CE, such as clinical trials and postmarket surveillance. Australia’s Therapeutic Goods Administration (TGA) demands a conformity assessment and clinical evidence submission. The Japan Pharmaceuticals and Medical Devices Agency (PMDA) implements pre- and postmarket regulations similar to those of the FDA.

The clinical validation process comprises preclinical analysis, which consists of laboratory-based studies to examine device functioning and safety, as well as clinical tests that are undertaken in many phases to evaluate device performance in a clinical environment. The entire process was divided into three phases: Phase I assessed safety and feasibility in a small sample of patients; Phase II gathered initial efficacy data in a larger group; and Phase III involved lengthy trials to verify effectiveness and safety. Protocol adherence involves following Standard Operating Procedures (SOPs) for consistent testing, adhering to Good Clinical Practice (GCP) standards, ensuring data integrity, and implementing risk management to identify and mitigate potential risks

### Impact on Clinical Decision Making

ML technology is transforming PoCT devices by significantly enhancing diagnostic accuracy, minimizing diagnostic times and improving patient care. These devices provide crucial insights to medical professionals by rapidly and precisely analyzing vast amounts of data, resulting in better clinical decisions and improved patient outcomes.

In cardiovascular treatment, these devices excel at recognizing complicated trends in electrocardiogram (ECG) data, detecting small anomalies indicative of early stage cardiac disease that humans might not recognize. Similarly, in dermatology, ML improves PoCT devices by assessing skin lesions. ML systems trained on large data sets discern between benign and malignant lesions with high accuracy, allowing for early identification of skin cancer and minimizing the need for intrusive biopsies.^144,145^

In infectious disease management, these technologies evaluate patient samples quickly, providing results in minutes rather than hours or days. Furthermore, these algorithms automate the interpretation of test data, relieve healthcare professionals of tedious analysis responsibilities. For example, in the treatment of diabetes, AI-ML-assisted glucose monitors instantaneously evaluate blood sugar levels and provide real-time insulin dose suggestions, improving efficiency and patient care.

ML algorithms use historical and real-time patient data to forecast disease development and possible effects. For example, in chronic disease management, such as chronic obstructive pulmonary disease (COPD), these devices screen patients and predict exacerbations, allowing healthcare providers to respond immediately and change medication strategies accordingly. Furthermore, ML algorithms tailor recommendations for treatment based on unique patient data, improving precision and effectiveness. In oncology, this technology analyzes genetic information from tumor samples and suggests individualized chemotherapy regimens, boosting therapeutic success while decreasing side effects.^115^

ML-assisted PoCT devices have significantly improved clinical decisions and patient outcomes in various scenarios. In diabetes care, continuous glucose monitors (CGM) with ML features predict glucose trends, minimizing hypoglycemia episodes and improving glycemic control.^146^ For sepsis, analysis of blood samples allows for prompt identification and treatment, lowering fatality rates.^147^ ML-enhanced cardiovascular ar care devices analyze ECG, blood pressure (BP), and cholesterol to detect cardiac events and atrial fibrillation in advance, reducing strokes and increasing patient outcomes. This preventative approach minimizes hospitalizations and allows for immediate responses and individualized patient care based on real-time data analysis.^148^ In oncology, devices use genetic profiling to tailor cancer therapy by assessing tumor biomarkers and mutations. The algorithms anticipate treatment responses to medications such as chemotherapy and immunotherapy for each individual patient based on genetic data, enabling doctors in choosing the most effective treatments, increasing treatment efficacy and survival rates.^149^ During infectious disease outbreaks such as the COVID-19 pandemic, these devices were critical in managing the outbreak by providing an early, accurate diagnosis, permitting successful containment, and maximizing utilization of resources. Overall, these improved technologies result in more accurate, timely, and individualized patient treatment, which significantly improves healthcare delivery.

### Integration with Clinical Workflow

To facilitate the successful integration of ML-assisted PoCT devices into existing clinical processes, it is important to address concerns about seamless integration, minimal interruption, and clear benefits. Here is an in-depth overview of how this technology can be smoothly integrated by focusing on user interface design, ease of use, and complementing traditional diagnostic methods.

Effective user interface design is essential for successfully integrating these devices into healthcare systems. The interface should be straightforward and easy to use with clear instructions and visual cues to reduce the process of learning and allow professionals to quickly become adept. Advanced customized dashboards with large, easy-to-read buttons and options enhance usability by allowing medical professionals to personalize the interface to their preferences and regularly perform tasks, which can considerably improve efficiency. Extensive and ongoing online or offline training programs, which include hands-on practical experience, simulated situations, and detailed user manuals, are required to familiarize healthcare providers with the new technology. Continuous support can occur via online resources, help desks, and regular software updates and additional accessories that incorporate feedback from user and new feature.^150^

To ensure ease of use, these devices should be compatible with existing electronic health record (EHR) systems that allow for automatic data transmission and decrease the need for human data entry. This can be achieved by using standard communication protocols (HL7 or FHIR) to facilitate seamless data sharing between PoCT devices and the hospital information technology systems. Devices should be designed for simple installation and calibration to reduce downtime and allow medical professionals to focus on patient care. Automated data collection and reporting capabilities enable results that are promptly and precisely entered into patient records, streamlining the workflow. Furthermore, the devices should be portable and lightweight, allowing physicians to use them conveniently at the patient’s or in other areas of the hospital. To provide continuous functioning throughout critical periods and prevent interruptions, these devices should have a prolonged battery backup, increasing their practicality as well as reliability in many clinical scenarios.^115^

To enhance seamless integration and diagnostic capabilities, devices must provide quick results, allowing for faster clinical decisions in emergencies and monitoring of chronic medical conditions. These can be used for preliminary screenings and routine monitoring, while standard lab tests can be saved for analysis that is more complex or for result confirmation. A sequential diagnosis approach should be used, with initial testing using PoCT devices followed by conventional methods if additional investigation is required. In addition, regular tests such as blood glucose and electrolytes reduce the load on centralized laboratories, allowing them to concentrate on more specialized tests. This approach not only increases workflow efficiency but also enables the early diagnosis of problems, resulting in prompt treatments and perhaps decreasing the need for additional conventional testing.

Addressing medical professional concerns about ML-assisted PoCT devices involves ensuring their dependability and accuracy through extensive validation studies and thorough quality control methods. Highlighting time-saving benefits, such as immediate outcomes and integrated decision support, improves workflow efficiency and clinical decision making. Clinicians can be certain that their patient data are secure by emphasizing compliance with data security standards and using strong encryption technologies. These measures collectively build confidence in the integration of modern diagnostic techniques into clinical practice.^150^

Implementation pilot programs in selected departments were implemented to evaluate device integration, receive feedback, and make necessary changes prior to full-scale rollout. Continuous feedback loops with doctors were created to provide continual communication to address complaints, improve functionality, and ensure that devices meet clinical needs efficiently. Continual improvements were prioritized by releasing regular software upgrades that include user feedback and technological advancements. Devices and accompanying algorithms were adapted to new clinical guidelines and rising diagnostic standards and regulations, retaining their relevance and efficacy in changing healthcare settings.^151^

### Patient-Centric Design and Usability

Designing ML-assisted PoCT devices with a patient-centric approach is crucial to ensure their effectiveness, compliance, and accuracy, particularly for at-home testing scenarios. Here are the key features and considerations.

Patient-centric approach in PoCT devices has significance importance, especially improving patient comfort, use, and accessibility. To minimize the anxiety and discomfort associated with testing, these devices should favor noninvasive or minimally invasive approaches. Furthermore, simplified user interfaces with straightforward instructions, audio and video graphics, and easy navigation can make the process easier and less daunting for the patients. Automating operations from sample collection to result interpretation improves usability by reducing human error and making the device accessible to people with diverse levels of technical proficiency. The devices should be portable, compact, and lightweight for convenient transportation and usage at home, permitting regular monitoring without the need for frequent trips to the hospital. Accessibility is another most significant factor, which can be achieved by keeping costs as low as possible through scalable manufacturing, ensuring that the devices are accessible to a broader population, especially those in resource-limited settings.^9,115^

**Figure 12:**
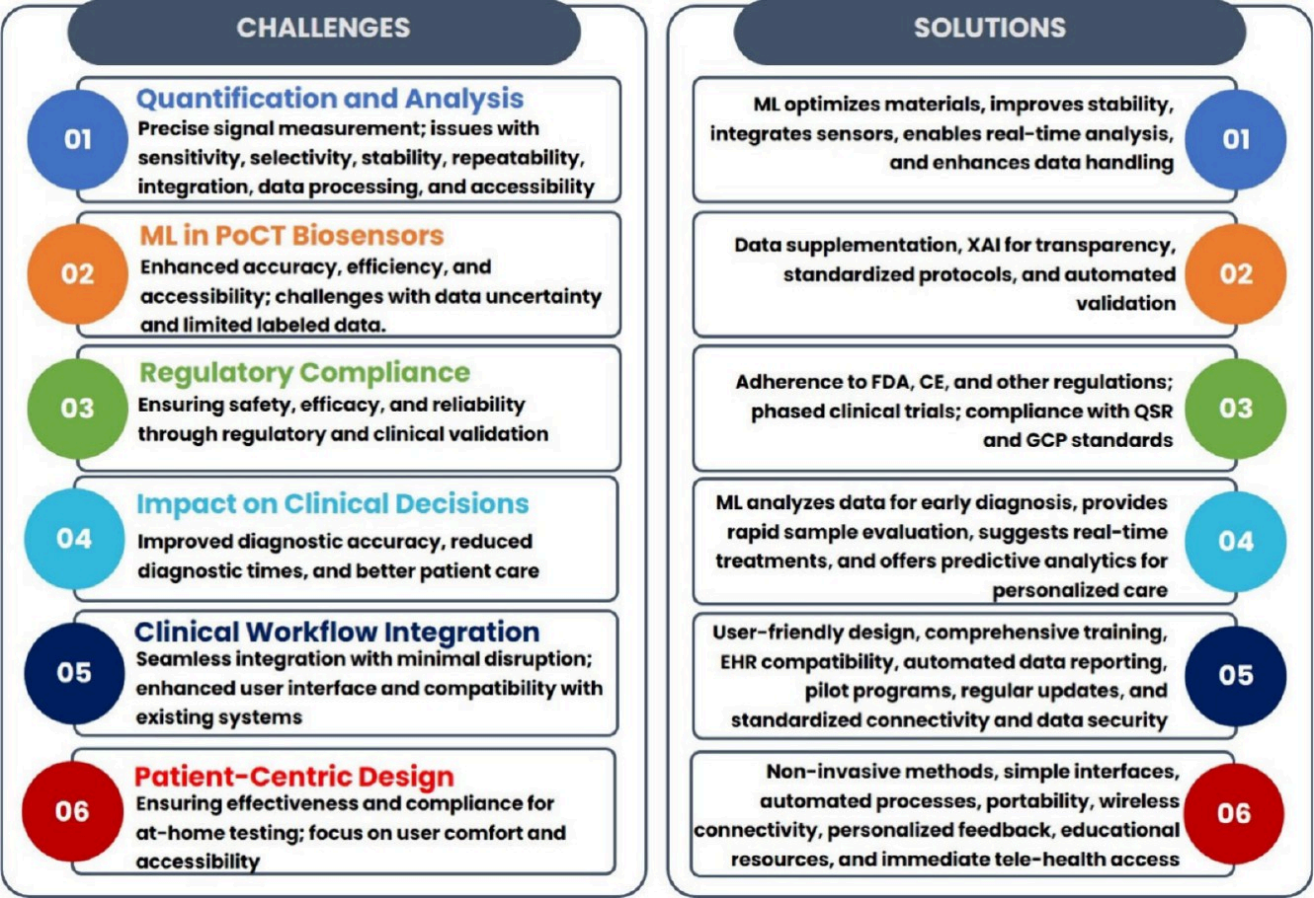
Diagrammatical representation of challenges associated with PoCT devices and future scope for exploring opportunities.

For effective at-home testing, devices include wireless connectivity and data integration, customization and adaptation, and education assistance. The integration with smartphones or other devices via Bluetooth or Wi-Fi provides easy data delivery to medical professionals for remote monitoring. Personalized user feedback-based systems on individual health data tracker and trends, enabled by ML algorithms, provide individualized health insights and suggestions, while adaptive interfaces that help to modify based on user preferences and past interactions can improve accessibility and patient engagement. Additionally, providing educational information within the device’s application can help users understand their diseases better and manage their health more effectively. Immediate and automatic access to telehealth and customer care services from the device can provide quick expert support when needed. To ensure clinicians of the effectiveness and reliability of ML-assisted PoCT devices, several key features are essential. Ensuring compliance and precise self-monitoring requires extensive validation and testing of ML algorithms to produce reliable and accurate outcomes, which are crucial for preserving physician faith. Features that record and remind patients about testing schedules and protocol adherence promote regular use, as well as accurate data collection. Continuous user feedback techniques, such as user-friendly error reporting systems, allow users to report problems quickly, which increases the reliability of the device. Collecting and evaluating user feedback can help lead to continuing improvements in device design and functionality. In addition, acquiring the relevant regulatory authorizations, such as FDA or CE certification, ensures that the device fulfills established health and safety requirements. Diagrammatical representation of challenges associate with ML assisted PoCT devices and future scope for exploring opportunities is shown in Figure 12.

## Conclusion

In contrast to a conventional laboratory-based diagnosis, PoCT typically shows lower reliability and accuracy. The inclusion of ML techniques into PoCT presents a promising pathway for improving the biosensor precision and dependability in real sample assessments. Leveraging smartphone applications equipped with ML algorithms could serve as a compelling solution for the direct interpretation of PoCT biosensor results. In addition, in light of the growing demand for accurate, versatile, and reliable sensors, biosensors with ML capabilities are well positioned to satisfy a wide range of needs in the healthcare industry. Therefore, enhancing the capabilities of next-generation medical technology requires incorporation of ML into portable biosensors and other health monitoring devices. This development could greatly accelerate the use of PoCT biosensors for self-testing or at-home applications.

## Vocabulary Section

Point-of-care-testing (PoCT): Refers to medical diagnostic testing performed at or near the site of patient care, providing immediate results to facilitate rapid clinical decision-making.

Biosensors: Biosensors are analytical devices that combine a biological component with a physicochemical detector to selectively and sensitively measure specific substances.

Healthcare: It refers to the organized provision of medical services, including diagnosis, treatment, and prevention of diseases, to maintain or improve an individual’s health.

Machine Learning (ML): ML is a branch of artificial intelligence that involves the development of algorithms that enable computers to learn from and make predictions or decisions based on data.

Diagnosis: Is the process of identifying a disease or medical condition based on a patient’s symptoms, history, physical examination, and often, diagnostic tests
